# A prospective open label 2–8 year extension of the randomised controlled ICON trial on the long-term efficacy and safety of occipital nerve stimulation in medically intractable chronic cluster headache

**DOI:** 10.1016/j.ebiom.2023.104895

**Published:** 2023-11-25

**Authors:** Roemer B. Brandt, Leopoldine A. Wilbrink, Ilse F. de Coo, Joost Haan, Wim M. Mulleners, Frank J.P.M. Huygen, Erik W. van Zwet, Michel D. Ferrari, Rolf Fronczek, ICON study group, Michel D. Ferrari, Leopoldine A. Wilbrink, Ilse F. De Coo, Patty G. Doesborg, Eveline C. Bartels, Erik W. Van Zwet, Frank J.P.M. Huygen, Wim M. Mulleners, Erkan Kurt, Robert T.M. Van Dongen, Onno P.M. Teernstra, Peter J. Koehler, Geert H. Spincemaille, Frank Wille, Katja Burger, Joost Haan, Emile G.M. Couturier, Jan Willem Kallewaard, Peter H. Veltink, R. Buschman

**Affiliations:** a Department of Neurology, Leiden University Medical Center, Leiden, the Netherlands; b Department of Neurology, Zuyderland Hospital, Heerlen, the Netherlands; c Department of Medical Rehabilitation, Treant, Emmen, the Netherlands; d Department of Neurology, Alrijne Hospital, Alphen a/d Rijn, the Netherlands; e Department of Neurology, Canisius-Wilhelmina Hospital, Nijmegen, the Netherlands; f Department of Anaesthesiology, Erasmus MC, Rotterdam, the Netherlands; g Department of Biomedical Data Sciences, Leiden University Medical Centre, Leiden, the Netherlands

**Keywords:** Cluster headache, Medically intractable chronic cluster headache, Occipital nerve stimulation, Neuromodulation

## Abstract

**Background:**

We demonstrated in the randomised controlled ICON study that 48-week treatment of medically intractable chronic cluster headache (MICCH) with occipital nerve stimulation (ONS) is safe and effective. In L-ICON we prospectively evaluate its long-term effectiveness and safety.

**Methods:**

ICON participants were enrolled in L-ICON immediately after completing ICON. Therefore, earlier ICON participants could be followed longer than later ones. L-ICON inclusion was stopped after the last ICON participant was enrolled in L-ICON and followed for ≥2 years by completing six-monthly questionnaires on attack frequency, side effects, subjective improvement and whether they would recommend ONS to others. Primary outcome was the change in mean weekly attack frequency 2 years after completion of the ICON study compared to baseline. Missing values for log-transformed attack-frequency were imputed for up to 5 years of follow-up. Descriptive analyses are presented as (pooled) geometric or arithmetic means and 95% confidence intervals.

**Findings:**

Of 103 eligible participants, 88 (85%) gave informed consent and 73 (83%) were followed for ≥2 year, 61 (69%) ≥ 3 year, 33 (38%) ≥ 5 years and 3 (3%) ≥ 8.5 years. Mean (±SD) follow-up was 4.2 ± 2.2 years for a total of 370 person years (84% of potentially 442 years). The pooled geometric mean (95% CI) weekly attack frequency remained considerably lower after one (4.2; 2.8–6.3), two (5.1; 3.5–7.6) and five years (4.1; 3.0–5.5) compared to baseline (16.2; 14.4–18.3). Of the 49/88 (56%) ICON ≥50% responders, 35/49 (71%) retained this response and 15/39 (38%) ICON non-responders still became a ≥50% responder for at least half the follow-up period. Most participants (69/88; 78% [0.68–0.86]) reported a subjective improvement from baseline at last follow-up and 70/88 (81% [0.70–0.87]) would recommend ONS to others. Hardware-related surgery was required in 44/88 (50%) participants in 112/122 (92%) events (0.35 person-year^−1^ [0.28–0.41]). We didn't find predictive factors for effectiveness.

**Interpretation:**

ONS is a safe, well-tolerated and long-term effective treatment for MICCH.

**Funding:**

The 10.13039/501100003246Netherlands Organisation for Scientific Research, the 10.13039/100009647Dutch Ministry of Health, the NutsOhra Foundation from the Dutch Health Insurance Companies, and Medtronic.

**Research in context**

**Evidence before this study**

We searched PubMed on November 13th, 2023, with the keywords “chronic cluster headache”, “cluster headache”, and “occipital nerve stimulation” (ONS), without restrictions to language or publication year. Of the 171 items, we excluded reviews and publications that did not report attack frequency at least 24 months after implantation. In publications combining different headache types, only data from patients with cluster headache were extracted. All publications only included data from retrospective or prospective long-term recordings of open-label assessment of the effect of ONS in case series. There were no prospective follow-up studies of the long-term effect of ONS in participants of a randomised controlled trial.

We included 10 studies from 9 unique study populations of patients with medically intractable chronic cluster headache (MICCH) in this review. No formal meta-analysis was performed due to the large heterogeneity of the design, nature (retrospective versus prospective), outcome measures, follow-up time, inclusion criteria and results. Instead, balanced means and ranges across all studies are reported. In total, 293 unique participants are reported with a mean follow-up of 54 months (range 37–87).

Most studies were small (<30 patients) and often there were methodological issues such as selection bias, uncertain length of the baseline period, incomplete or no formal statistical assessment at all of outcome and adverse events, little or no information on the number of participants that were lost to follow up and how these were statistically handled, and data reported only at the last follow-up with no information on effects at earlier intervals.

All studies showed a reduction in attack frequency (mean 55%; median 50%, range 25%–95%). Detailed data on adverse events was lacking, but a mean of 58% (range 24%–80%) of participants experienced one or more (serious) adverse events during follow-up.

**Added value of this study**

This study provides a prospective detailed follow-up (range 2–8.5 years) of the long-term efficacy and safety of ONS in patients with MICCH who had participated in the only randomized, dose-controlled study of the effects of ONS in MICCH to date (ICON trial). Both participants who had improved at the end of the randomised trial and those who had not improved were included in the follow-up, providing important information on whether early improvement persisted and whether delayed improvement could occur with continued treatment. Moreover, compared to existing studies, this study had minimal selection bias, a long specified baseline of 3 months, full evaluation of outcome and adverse events at regular half-yearly intervals up to 8.5 years, and detailed information on the number of participants who were lost to follow up and why.

The mean follow-up was 4.2 ± 2.2 years for a total of 370 person-years. Objective and subjective sustained efficacy were high. More than two-third of the ≥50% responders at the end of the ICON study retained this response. Moreover, during the follow-up period, more than one-third of the non-responders converted to a ≥50% responder for at least half the follow-up period. At the last follow-up, subjective improvement from baseline was reported by 78% of the participants and 81% would recommend ONS to other patients with MICCH. Hardware-related serious adverse events (SAE; formally defined as “serious” solely because short hospitalisations were required for replacements) occurred in 55% of participants with a SAE rate of 0.37 person-year.

**Implications of all the available evidence**

ONS offers long-term and well-tolerated improvement in MICCH and may also be considered for patients with CCH who respond only sub-optimally to standard medical treatment but do not yet meet the strict criteria of MICCH. Improved ONS devices and stimulation protocols currently under development may confer even better effects but that remains to be demonstrated.

## Introduction

Medically intractable chronic cluster headache (MICCH) is the most extreme and disabling form of cluster headache, in which patients continue to have frequent, often daily, attacks despite a variety of standard prophylactic medication.^1^, ^2^, ^3^, ^4^, ^5^

We showed in the randomized double-blind ICON study that both 100% and 30% electrical dose occipital nerve stimulation (ONS) were safe, well tolerated and reduced attack frequency by an average of 50% in people with MICCH for at least 48 weeks.^6^^,^^7^ Because active ONS causes paresthesia and thus would lead to deblinding when compared with inactive sham stimulation, we compared high versus low electrical dose ONS causing similar paresthesia, thus preserving blinding. We assumed and told the ICON participants that the 30% stimulation would be ineffective or much less effective, which ultimately proved to be false. The primary endpoint was “attack reduction compared to baseline”, which was met in both groups. Since there was no difference between treatment groups, a placebo response cannot be formally ruled out but is considered highly unlikely because of the observed abrupt, marked and sustained improvement in patients with long-term severe MICCH in a study with confirmed blinded treatment.^6^ The 130 participants in this study had not responded to, were intolerant of or had a contraindication to verapamil *and* lithium, as well as at least one of the following medications: methysergide, topiramate or gabapentin.^1^^,^^4^ The mean attack frequency and intensity decreased within a few weeks of starting ONS from about 16 to 8, respectively, at baseline to about 7 and 6 after the first double-blind 24-week study period. After the second 24-week study period in which open-label individually optimised ONS was given the mean attack frequency and intensity remained stable at about 8 and 5, respectively. At the end of the ICON study, the overall median relative reduction in attack frequency was 50% and in attack intensity 32%. Half of the patients had a ≥50% reduction in attack frequency. Treatment was well tolerated and more than 90% of patients were satisfied or very satisfied.

Previous retrospective^8^, ^9^, ^10^ and prospective^11^, ^12^, ^13^, ^14^, ^15^, ^16^, ^17^, ^18^, ^19^ open-label observational studies had shown promising results on the longterm,^20^ although in two smaller studies the effectiveness of ONS actually decreased over time.^15^^,^^17^ However, these observational studies all had a number of important methodological concerns, including mostly small numbers of participants, short or ill-defined baseline periods and duration of follow-up, ill-defined outcome measures and inclusion criteria, lack of information on efficacy and adverse events in participants lost to follow-up and how this was handled statistically, and incomplete analyses of the effectiveness and side effects.

In this structured follow-up study, we prospectively and in detail evaluated the long-term effectiveness and safety of ONS for at least two years in 88 Dutch participants of the ICON trial (L-ICON). Most participants could be followed for much longer, some even for more than eight years. In particular, we evaluated: (i) whether responders remained responders over time; (ii) whether satisfied participants remained satisfied; and (iii) whether non-responders and/or dissatisfied participants could still become responder and/or satisfied.

## Methods

### Ethics

Written informed consent was obtained from all participants and the study protocol was approved by the ethical committee of the LUMC (METC-LDD; Protocol number P10.016).

### ICON trial

The parent study (ICON trial) was an investigator-initiated, international, multicentre, randomised, double-blind, phase 3, electrical dose-controlled clinical trial. After 12 weeks of baseline observation, participants were randomised to 24 weeks of occipital nerve stimulation at either 100% or 30% of the individually determined range between paresthesia threshold and near-discomfort (double-blind phase of the study). In weeks 25–48, participants received individually optimised open-label ONS.^15^^,^^17^

At the beginning of the ICON study, it was assumed that 30% stimulation was clinically ineffective, yet caused the same paresthesia as the 100% stimulation and thus was useful as a blinded sham/placebo comparison. At the end of the study, however, 30% stimulation was found to be as effective as 100% stimulation. Blinding remained throughout the first double-blind phase of the study.

### Patients and questionnaires

For logistic reasons, only Dutch patients who had completed the full 48-week ICON study were invited for prospective structured follow-up every 6 months for at least two years but if possible longer.

ICON study participants were asked to participate and enrolled in the L-ICON follow-up study immediately after completion of the ICON study, 48 weeks after ONS implantation. Meanwhile, the ICON study continued to enrol new patients until 3 December 2017. Therefore, initial ICON participants could start earlier in L-ICON and be followed longer than ICON participants enrolled later in the ICON study. Inclusion for the L-ICON study was stopped after the last ICON participant was enrolled in L-ICON and followed for ≥2 years (i.e. ≥3 years after ONS implantation).

Participants who consented had to complete two web-based questionnaires every six months. Unless they had explicitly withdrawn, the participants were reminded to complete the questionnaires six and two weeks before and two and six weeks after every six-month deadline. The six-monthly reminders were sent regardless of whether or not they had submitted the questionnaires six months earlier.

One questionnaire included questions on: (i) neurostimulator use; (ii) side effects and complications; (iii) mean weekly attack frequency over the past six months; (iv) whether participants experienced improvement and if so, an estimated percentage of improvement; and (v) whether patients would recommend this treatment to a similar patient on a five-point scale. The other questionnaire consisted of the MOS *36-Item Short-Form Health Survey* (SF-36) to measure quality of life in ten subscales.^21^, ^22^, ^23^

### Statistics

Because participants in the LICON follow-up study were each enrolled immediately after completion of the ICON study (i.e. 48 weeks after ONS implantation), while the ICON study was still recruiting new patients until December 3rd 2017, the initial LICON participants (who started the ICON study already in 2013) had a much longer (potential) follow-up than those enrolled later (e.g. only in 2016). We therefore calculated the maximal potential follow durations for each individual participant and the entire group. First we calculated the potential maximal follow-up duration for each individual participant from the time they were included for the L-ICON follow-up study (i.e. immediately after they completed the ICON study 48 weeks after ONS implantation) to the end of the L-ICON follow-up study. Then, we calculated the total potential follow-up duration for the entire group by summing all individual potential follow-up durations and adding them together for all participants.

Descriptive analyses were used to visualize response after ONS implantation. Furthermore, missing values for attack frequency and three SF36 items (mental health sum score, general health and physical health sum score) were imputed until 5 years after completion of the ICON trial (after the end of the open label extension, i.e. 48 weeks after implantation). Variations in the imputed datasets were analysed and pooled data from five different imputed datasets was used. Age, body mass index (BMI), sex, weekly attack frequency, mental health sum score, physical health sum score and general health score were used as predictors with predictive mean matching. Because of the low number of data points from 5 years after ICON trial completion, data was imputed until 5 years after completion of the ICON trial. The distribution of the attack frequencies was skewed. Therefore, we used the logarithm of the weekly attack frequency in the analyses and the geometric mean for the representation of the pooled data. Data is depicted with a 95% confidence interval (CI) around the pooled geometric or arithmetic mean. A Kaplan Meier survival analysis was performed for time to first treatment success, defined as ≥ 50% or ≥30% reduction in attack frequency. Participants were censored if the event had not occurred 5 years after ICON trial completion. A binary logistic regression model was used to predict a yes/no ≥ 50% reduction in attack frequency at 2 years after ICON trial completion. Sex, restlessness during attacks and ≥50% response in week 1–4, after implantation were used as categorical predictors. Age, mean weekly attack frequency at baseline and number of autonomic symptoms were used as continuous predictors. Adverse events and serious adverse events were depicted using a rate point estimate with a 95% confidence interval. As in the ICON study, serious adverse events were defined according to EN ISO 14155-1, which implies that surgical interventions and hospital admissions labelled as serious adverse events, irrespective of the nature of the hospital admission.

### Outcomes

The primary outcome was the change in mean weekly attack frequency 2 years after completion of the ICON study (i.e. 3 years after ONS implantation) compared to baseline (i.e. the three months before ONS implantation). Secondary outcomes were (i) Change in mean attack frequency from baseline after 1, 2 and 5 years (ii) whether non-responders (i.e. <50% or <30% reduction in attack frequency from baseline); at the end of the ICON study became responders during long-term follow up; (iii) occurrence of adverse events; (iv) mean SF-36 scores; (v) predictive capacity of sex, age, restlessness, mean weekly attack frequency at baseline, number of autonomic symptoms and ≥50% response in week 1–4 after implantation for efficacy at two years and (vi) subjective improvement and willingness to recommend this treatment to other patients.

### Role of the funding source

The funders had no role in the study design; in the collection, analysis, and interpretation of data; in the writing of the report; and in the decision to submit the paper for publication.

## Results

### Participants

**Fig. 1:**
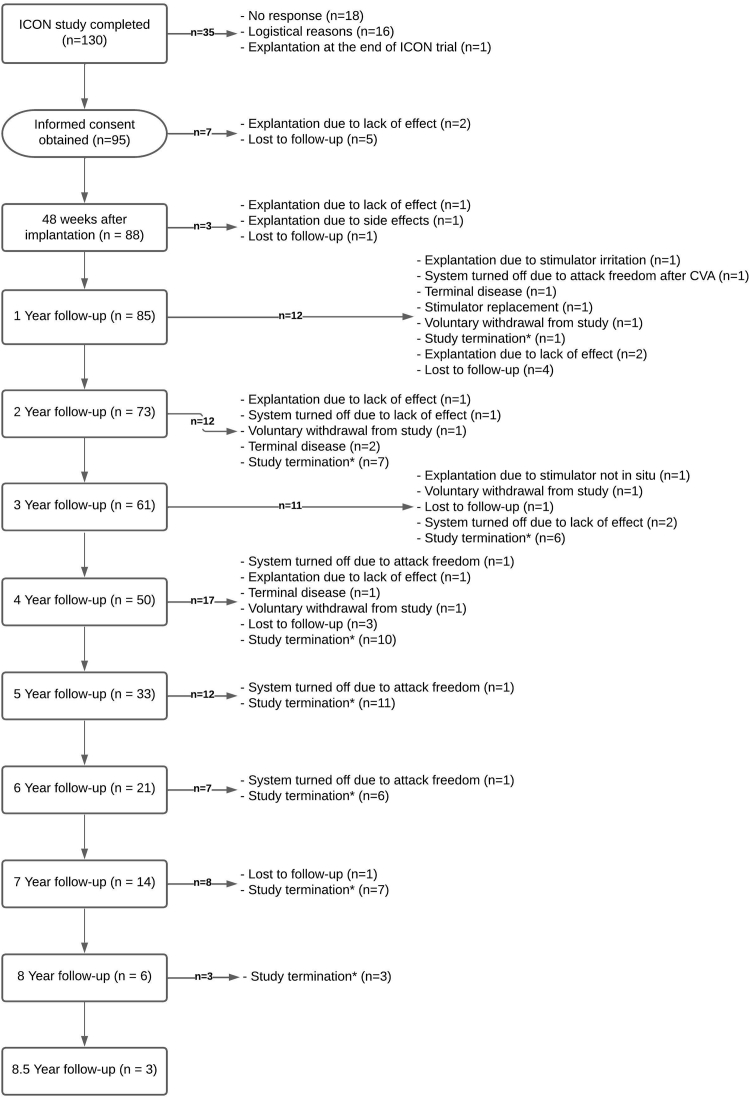
Study flowchart. ∗Study termination: Inclusion for the L-ICON study was stopped after the last ICON participant was enrolled in L-ICON and followed for ≥2 years (i.e. ≥3 years after ONS implantation).

As shown in Fig. 1, of the n = 130 participants in the ICON study n = 119 were from the Netherlands and were eligible to participate in L-ICON. Of these, n = 16 were excluded because they had not completed the entire 48 weeks of the ICON study (n = 8) or could not be approached for logistical reasons (n = 8). Therefore n = 103 ICON participants were invited to participate in the L-ICON study of whom n = 95 (92%) gave written informed consent and n = 88 (85%) actually started the follow-up study between September 1st 2012 and January 14th 2019 in four centers in the Netherlands. Active follow-up was stopped in December 20th 2020, when all participants had at least a 2-year follow-up or were censored due to drop-out. Mean (±SD) follow-up was 4.2 ± 2.2 years, for a total of 370 person years.

No correlation was observed between ICON participants with or without a 50% attack reduction at the end of the ICON trial and participation in the L-ICON (Odds ratio 0.95 [0.40–2.24], p = 0.902).

**Fig. 2:**
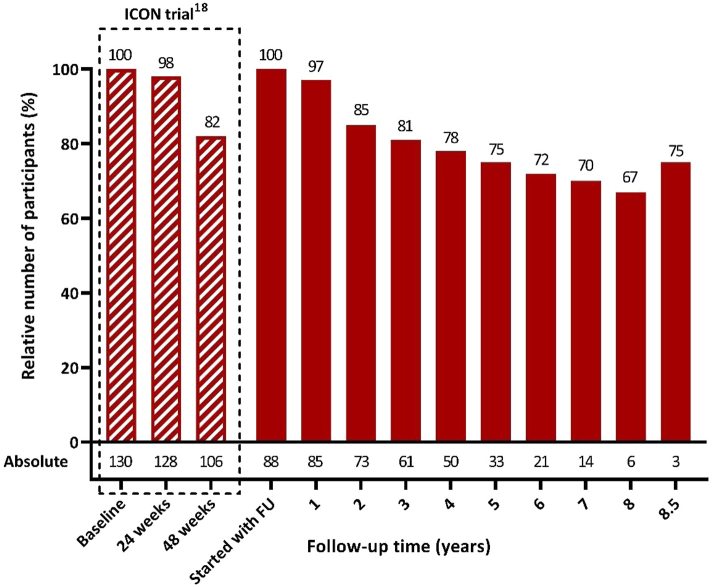
**Number of active participants**. Because participants in the LICON follow-up study were each enrolled immediately after completion of the ICON study (i.e. 48 weeks after ONS implantation), while the ICON study was still recruiting new patients until December 3rd 2017, the initial LICON participants had a much longer follow-up than those enrolled later. The actual number of active participants is shown relative to the potential maximum number of participants for that period.

Due to the staggered inclusion of L-ICON participants, each time after they had completed the 48-week ICON study, and because follow up took place in parallel with the continuing ICON study, the duration of follow-up was longer for initial L-ICON participants than for those enrolled later. Active follow-up was ≥2 year in 73 (83%), ≥3 year in 61 (69%), ≥5 years in 33 (38%) and ≥8.5 years in 3 (3%) participants (Figs. 1 and 2). The mean (±SD) follow-up was 4.2 ± 2.2 years for a total of 370 person years, which is 84% of a potential maximum total duration of 442 person years. Missing data, also of non-responders, were imputed up to 5 years after completion of the ICON study. Follow-up was prematurely terminated in 34/88 (39%) participants, because of explantation (n = 8), turning off of the ONS device because of lack of efficacy (n = 3), lost to follow-up (n = 10), personal reasons (n = 4), death due to other disease (n = 4), or attack freedom (n = 3), or no effect but scared to stop the ongoing stimulation (n = 2).

Key baseline characteristics are summarised in Table 1. There were no differences for age, sex, attack frequency at baseline and at the end of the ICON study, and absolute and relative response at the end of the ICON study between participants and non-participants (data not shown).

**Table 1:** Baseline characteristics.

Mean age (years ± SD)	45.8 ± 13.7
Sex (n, % male)	60 (65.9%)
Smoking (n, %)	49 (53.9%)
BMI (median, IQR)	24.8 (22.0–29.8)
Median weekly attack frequency at baseline (IQR)	15.8 (9.7–24.7)
Median cluster headache duration (years, IQR)	8.0 (8.0–13.0)
Median time since diagnosis (years, IQR)	5.5 (3.0–10.0)
Median duration of chronic cluster headache (years, IQR)	4.0 (2.0–7.0)
Mean follow-up^a^ (years ± SD)	4.2 ± 2.2

a Follow-up started after completion of the ICON trial (48 weeks +10 days run-in phase after implantation).

### Attack-frequency

**Fig. 3:**
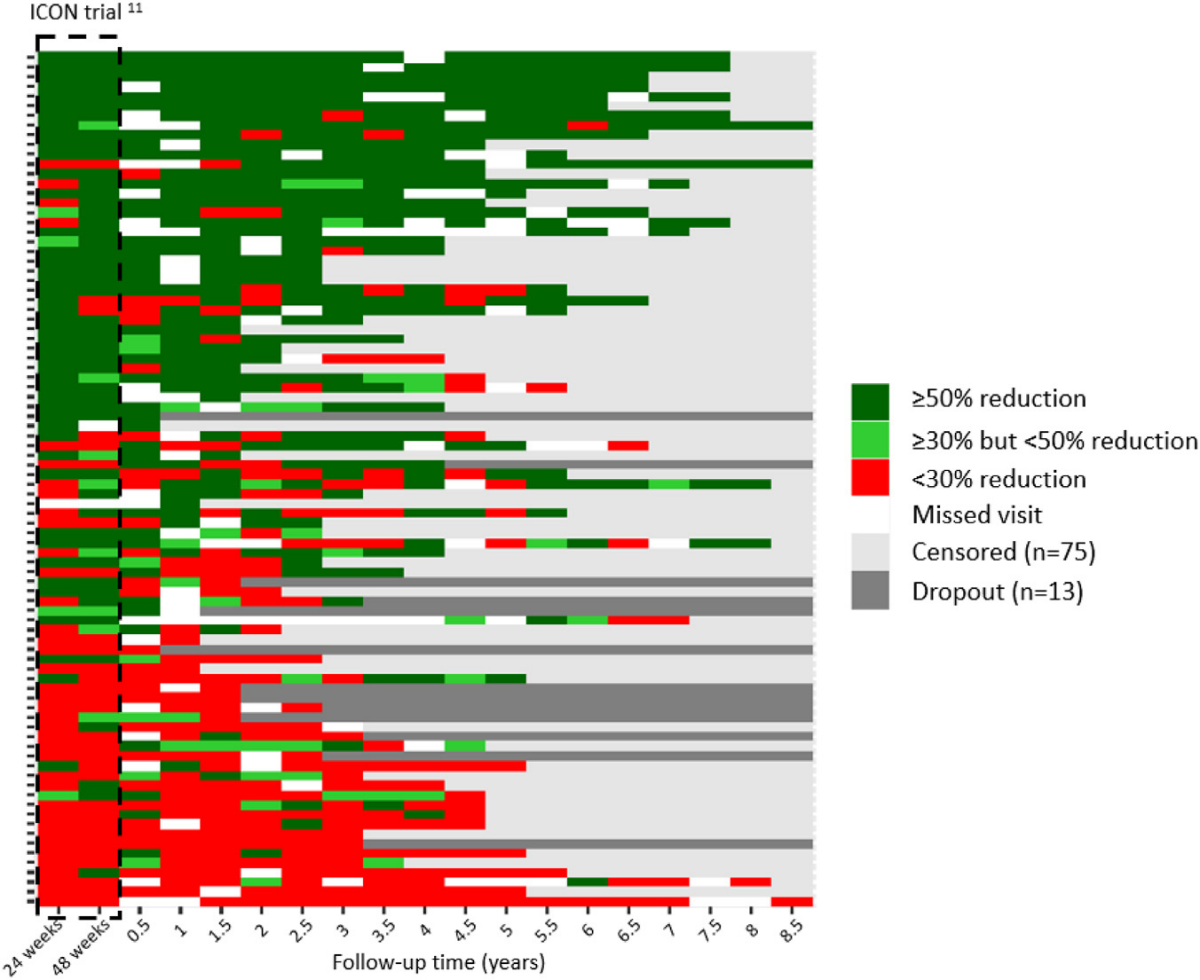
Heat map depicting each individual participants' response to ONS until 8.5 years after ICON study completion. Patients are sorted according to responder status with those on the top achieving the highest number of time points with positive responses. Individual follow-up duration is dependent on enrolment date. Explantation (n = 8), device turned off due to no effect (n = 3) and no effect (n = 2) have been marked as ‘dropout’ (n = 13). End of follow-up due to study ending (n = 57), lost to follow-up (n = 10), personal reasons (n = 4), death due to other disease (n = 4) or attack freedom (n = 3) have been marked as ‘censored’ (n = 75). Data at 24 and 48-week time point are from the ICON trial.^6^

Fig. 3 visualises the reduction in weekly attack-frequency for each individual horizontally from left to right for each 6-month follow-up period compared with baseline to the end of follow-up: ≥50% reduction (dark green), ≥30% but <50% reduction (light green), or <30% reduction (red). Each horizontal block represents an individual 6-month follow-up period. Participants are sorted vertically from top to bottom by the number of 6-month blocks in which a ≥50% response or a ≥30% but <50% response was observed minus the number of blocks in which a <30% response was observed. The more sustained the efficacy, the higher the participant appears in the figure.

Supplemental Fig. S1a and b shows the Kaplan Meier curve until the first time a participant showed a ≥50% (a) or ≥30% (b) attack reduction. All participants that had not achieved this reduction were censored at 5 years after the end of the ICON trial.

Of the 49/88 (56%) participants who had a ≥50% attack reduction at the end of the ICON study, 36/49 (73%) retained this response for at least half of the follow-up period. Of the 39/88 (44%) participants who were not a ≥50% responder at the end of the ICON study, 15/39 (38%) still became a ≥50% responder for at least half of the follow-up period. A total of 52/88 (59%) participants had a ≥50% response for at least half of the follow-up period.

Of the 57/88 (65%) ≥30% responders at the end of the ICON study, 47/57 (82%) retained this response for at least half of the follow-up period and of the 31/88 (35%) <30% responders at the end of the ICON study, 11/31 (35%) still became a ≥30% responder for at least half of the follow-up period and 4/31 (13%) even became a ≥75% responder.

Of the n = 36/88 (41%) who had a ≥75% response at the end of the ICON study, 24/36 (67%) retained this response for at least half of the follow-up period.

**Fig. 4:**
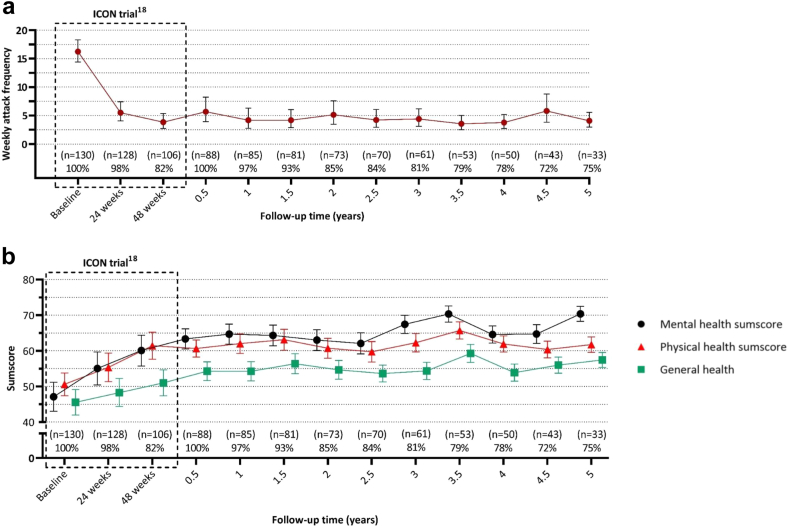
Evolution of pooled geometric mean score of (a) median weekly attacks and (b) three SF-36 items (mental health sumscore, general health and physical health sumscore) with 95% confidence interval and number of active participants as percentage of possible participants. Because participants in the LICON follow-up study were each enrolled immediately after completion of the ICON study (i.e. 48 weeks after ONS implantation), while the ICON study was still recruiting new patients until December 3rd 2017, the initial LICON participants had a much longer follow-up than those enrolled later. Both the actual number of active participants is shown and the number relative to the potential maximum number of participants for that period.

Fig. 4a and Supplemental Table S1a show a reduction in the pooled mean [*95% CI*] attack frequency after one (4.2; *2.8–6.3*), two (5.1; *3.5–7.6*) and five years (4.1; *3.0–5.5*) compared to baseline (16.2; *14.4–18.3*).

Similarly, Fig. 4b and Supplemental Table S1b show that the pooled mean [*95% CI*] of each of the three SF-36 scores remained improved during follow up after 1, 2, and 5 years.

### Subjective response and satisfaction

Most participants (69/88; 78% [68%–86%]) reported a subjective improvement from baseline at their last follow-up; 9/88 (10% [5%–19%]) reported no change and 4/88 (5%) a worsening. The remaining 6 (7%) could not answer the question since they had a broken stimulator (n = 2) or the stimulator was turned off for varying reasons (no more attacks (n = 1), no effect (n = 1), unknown (n = 2)).

Most participants would also recommend (70/88; 81% [70%–87%]) this treatment to other patients; the majority would even make a strong recommendation (52/70, 74% [48%–69%]). Only 2 (2%) participants would not recommend ONS and 14/88 (16%) did not have a (strong) opinion.

### Predictive factors for efficacy at 2 years

Sex, age, number of autonomic symptoms, disease duration, restlessness and attack frequency at baseline are presented in Supplemental Table S2.

### Adverse events

From the end of the ICON trial to the last follow-up visit, N = 202 serious adverse events (SAE) occurred in 63/88 (72%) participants (Supplemental Table S3). Of these n = 122 were hardware-related in 48/88 (55%) participants and n = 79 were non-hardware related in 40/88 (45%) participants. This corresponds to an overall SAE incidence rate of 0.62 person-year^−1^ [0.54–0.71], a hardware-related SAE incidence rate of 0.37 person-year^−1^ [0.31–0.45] and a non-hardware-related SAE incidence rate of 0.24 person-year^−1^ [0.19–0.30]. A total of 112/122 (92%) hardware-related SAEs required additional surgery in 44/88 (50%) participants, corresponding to an hardware-related additional surgery rate of 0.35 person-year^−1^ [0.28–0.41]. In total, 593 hardware-related adverse events (AE) were reported in 71/88 participants (81%), corresponding to an overall AE incidence rate of 1.83 person-year^−1^ [1.68–1.98].

## Discussion

In this study, we prospectively and structurally assessed the long-term effectiveness, safety and tolerability of ONS in 88 patients with MICCH who had initially completed the 48-week randomised, double-blind, electrically dose-controlled ICON trial. Both ICON responders and non-responders were followed for at least two years with a mean of four years and a total of 370 person-years which is 84% of a potential maximum total duration of 442 person years. After 3 years of follow-up, 60/88 (68%) participants were still active, and after five years, 32/88 (36%); three participants could be followed for 8.5 years. The majority of participants who were ≥50% responders at the end of the ICON study remained responder for most of the follow-up period, and more than one third of initial non-responders became ≥50% responders. Mean group attack-frequency and the SF-36 general health scores also remained improved for at least 5 years. Remarkably, most participants, even those in whom attack frequency did not significantly decrease, reported subjective improvement and would recommend ONS to other patients with MICCH. ONS was generally well-tolerated. Most SAEs were due to a short hospital stay for hardware replacement. The overall incidence per person-year for SAEs was 0.62, for hardware-related SAEs 0.37, for non-hardware-related SAEs 0.24, for additional hardware-related surgery 0.35 and for all non-serious AEs 1.83.

ONS is a relatively new, minimally invasive and reversible prophylactic treatment for MICCH. Small open-label case series^9^^,^^12^, ^13^, ^14^^,^^18^ and the randomised controlled ICON trial^6^^,^^7^ all showed very good short-term results. In the current prospective follow-up study, we demonstrate continued and mostly stable efficacy and safety for at least five years (Fig. 3). This is all the more striking when one considers that the patients in this study had been severely disabled and intractable for years. They all suffered from incessant severe attacks of cluster headache that did not respond to standard prophylactic medical treatment.

The results are in good agreement with those of other retrospective^8^ or prospective^10^^,^^11^^,^^13^^,^^16^^,^^17^ observational studies of ONS efficacy in MICCH. However, compared with our study, these were mostly smaller and unstructured, and had shorter or ill-defined duration of follow up and baseline, ill-defined outcome measures and inclusion criteria, and lack of information on the efficacy and adverse events in participants lost to follow-up and how this was handled statistically. Moreover, none of the other studies, like ours, was a prospectively planned, structured, open-label, long-term extension of a randomised, double-blind controlled trial.^6^^,^^7^

It was notable that even participants without an objective reduction in attack frequency reported subjective improvement and satisfaction, and continued ONS therapy. This was probably due to a reduction in attack severity and an improved response to acute and prophylactic medical treatment, as in the ICON study and other trials.^6^^,^^8^^,^^11^ Because intensity often varies across attacks and is therefore difficult to measure reliably, it was not monitored in this study.

As in other studies,^11^^,^^14^ a substantial proportion of initial non-responders (38%) became ≥50% responders over time (Fig. 3, Supplemental Fig. S1). We can only speculate about the reasons and mechanisms for this. Network plasticity (i.e. improved functional neuronal connectivity changes in the pain processing network) has been suggested as an explanation for a later improvement.^11^^,^^14^ However, in the ICON study, most improvement occurred within the first few weeks after implantation and almost no one improved in the open-label second half of the study in which the ONS settings were individually optimised. It is possible that in some patients 48 weeks is still too short a period to achieve network adaptation and longer periods are needed. However, if there has been no improvement within 2 years, the chances of improvement later on decrease dramatically.^24^

In the ICON study, we used non-rechargeable implantable pulse generators (IPGs) and hardware originally developed for epidural spinal cord stimulation, which increased the risk of fracture or dislocation of the leads when used for ONS. However, for necessary replacements during follow-up, we were able to use (i) rechargeable IPGs, reducing the need for surgical battery replacement; (ii) new, more flexible electrodes that adapt better to the shape of the skull, reducing the risk of fracture; and (iii) tined leads, reducing the risk of dislocation. The number of hardware-related additional surgeries was 0.35 per person-year, which is similar to other ONS studies.^6^^,^^25^ Two third of these additional surgeries were lead replacements and one-third were battery replacements. Importantly, no biological SAEs occurred and all ONS-related SAEs required only minor surgery with minimal hospitalization. However, it is important to realise that, according to the formal guidelines such events, even those requiring only minor surgery and a short hospital stay, should be classified as serious AEs. There were no unexpected SAEs associated with ONS. Although side effects were common, they were usually minor, such as neck stiffness and local pain, and ONS was well tolerated.

We found no significant predictors of long-term response, consistent with previous studies.^6^^,^^11^ However, due to the limited power, we cannot exclude possible predictive factors. Response to a previous greater occipital nerve block might be an interesting candidate as the mechanism of effect is likely to be similar, although this could not be confirmed in two previous studies.^8^^,^^26^

Important strengths of this follow up study include (i) the large sample size, with both responders and non-responders to 48 weeks ONS at the start of the follow up, minimising the risk of a large selection bias; and (ii) the long and structured prospective follow-up period with a high retention rate of 73/88 (83%) active participants at the end of the predetermined minimum follow-up of two years, i.e. three years after ONS implantation. In fact, the majority of participants in the L-ICON follow-up study were followed even longer than two years because they were included only after they had each completed the 48-week ICON study, the inclusion for the ICON study was spread over many years, and because the L-ICON follow-up took place in parallel with the ICON study. Of the 88 participants who started follow-up, 61 (69%) could be followed ≥3 years, 33 (38%) ≥5 years and 3 (3%) for as long as ≥8.5 years. Based on the actual, compared to the potential maximum duration of follow up–because not all participants started at the same time -, drop out accounted for only 16% of the total person years.

Apart from drop-out and loss-to-follow-up, events that reflect everyday practice and are unavoidable in this type of study, other limitations should be considered. Not all participants in the ICON study took part in the L-ICON follow up. A total of 19 participants from the ICON trial who did not participate in the L-ICON were missing completely at random (i) N = 11 did not live in the Netherlands and thus were not approached to participate in L-ICON for logistical reasons; and (ii) N = 8 were not approached because these patients had started the ICON study very late and therefore would have ended the ICON study very late; inclusion of these 8 patients would have delayed the end of the L-ICON study by at least two years. Therefore, their missingness should not have biased the results. Furthermore, no differences were observed between participants and non-participants in key demographic and clinical characteristics at baseline. Moreover, response rates during the ICON study did not differ between L-ICON participants and non-participants. Finally, response rates in the ICON study did not correlate with the likelihood of participating in the L-ICON study.

The main statistical analysis was performed for a follow-up of 5 years after completion of the ICON trial. Because of the research design (staggered inclusion), not everybody had an equally long follow-up, therefore missing data are unavoidable. The reasons for missingness should be considered very carefully as non-random missingness could bias the results. Most of the missing data after 5 years of follow-up is due to the fact that, because of the staggered inclusion, these participants had not yet reached 5 years of follow-up after completion of the ICON trial when the study ended (n = 24 cases, Fig. 1). These data can therefore be regarded as “missing completely at random”. In the event of study termination due to lack of effect or attack freedom, it is reasonable to assume that the missingness can be explained by reasons about which we have full information (i.e. attack frequency and quality of life). We therefore assume that the reason for missingness is likely present in the data points before the missing data (lack of effect or complete remission). Accordingly, we feel that the missingness should be labelled as missing at random rather than not at random.

Moreover, in an effort to address these missing data and minimise selection bias and biased data loss, we used the full observations as a template for the incomplete observations by using multiple imputations that allowed us to use the data from all ICON trial participants, including those who showed no effect in or completed the ICON trial. By assuming that the missingness of the data is (completely at) random, it is reasonable to extrapolate the measurements. The assumption is that if measurements are stopped in a participant, the future data will behave similarly to the data of similar participants who did remain in the study. To illustrate the robustness of our data, we also performed an analysis without imputed data which showed similar data to those with imputations (Supplementary Fig. S3), reinforcing our results. If the reason for missingness was due to a sudden increase or decrease in attack frequency that we did not record, the data would be missing not at random (MNAR). We consider this unlikely because of the stable observations during follow-up, but we cannot rule out the possibility either. There is no way to correct for MNAR, but we performed a sensitivity analysis in which we considered worst case and best case scenarios that would bracket the truth. We generated these scenarios by increasing and decreasing all previously imputed attack frequencies in the participants that were missing data for other reasons by 50%. We then ran our analysis again and found a similar result (Supplementary Fig. S4).

As with all headache studies, we had to rely on self-reported data on attack frequency and adverse events. However, we believe the data are accurate and complete. Although prospective recording of attacks immediately after each attack using an electronic diary is considered the most optimal and reliable way to measure attack frequency, it is also the most burdensome for patients.^24^ This, in turn, may make the results less reliable. Since retrospective registration of attacks once a week is much less burdensome for patients and we have shown that the accuracy of retrospective versus prospective registration of attacks is in fact very similar,^27^ we believe that the method we used for the L-ICON study (retrospective registration of attack frequency once a week) is reliable and accurate. The recording of hardware-related serious adverse events was externally validated. Every hardware-related serious adverse event was documented in the database. Non-hardware-related SAEs and all non-serious AEs were self-reported. The questionnaire explicitly asked about the occurrence of hardware-related AEs. If participants had experienced any hardware-related AE, it is highly unlikely that they did not report them since they were explicitly asked about them.

Finally, all participants previously did not respond (sufficiently) to an optimal dose of, or were intolerant of and/or had a contraindication to verapamil and lithium, as well as at least one of the other recommended treatments for cluster headache: methysergide, topiramate or gabapentin. During the L-ICON follow-up study, all participants received individually optimised treatments and were allowed to use any prophylactic drug in addition to ONS. However, these drugs had previously proven ineffective for these patients and no new drugs had become available during the ICON and L-ICON studies. We therefore conclude that the observed effects were due to ONS and not to other concurrent prophylactic medications. Because of the extremely high disease burden, several neuromodulatory options with a wide range of targets such as sphenopalatine ganglion block and stimulation, vagal nerve stimulation, greater occipital nerve blocks and stimulation, and even deep brain stimulation (DBS) have been explored with mixed results.^28^, ^29^, ^30^, ^31^, ^32^, ^33^ However, these therapies are often only used as a transitional treatment or, in the case of DBS, associated with high risk. ONS is an effective and well-tolerated treatment for MICCH, providing significant and sustained improvement for at least five years. In future studies, we should study the efficacy, safety and economic value of the even more advanced and less invasive forms of ONS currently under development and compare them with those of existing standard medical treatments in MICCH and sub-optimally drug-responsive common chronic cluster headaches.

## Contributors

Study design (conceptualization and methodology): LAW, JH, WM, FJPMH, EWZ, MDF, ICON study group; Data collection and curation: RBB, LAW, IFC; Data analysis and formal analysis: RBB, EWZ; Writing—original draft: RBB, MDF, RF; Writing—review and editing: RBB, LAW, IFC, JH, WM, FJPMH, EWZ, MDF, RF; Supervision: MDF, RF.

RBB and EWZ have verified the underlying data. All authors commented on the manuscript and approved the final version of the manuscript.

## Data sharing statement

The data sets used and/or analysed during the present study are available from the corresponding author on reasonable request.

## Declaration of interests

WM reports honoraria from Novartis, Teva, AbbVie, Lundbeck and Lilly, and consultancy and lecture fees from Lilly; RF reports consultancy and lecture fees from Novartis, Lundbeck, AbbVie, Lilly and TEVA, and independent support from the Dutch Brain Foundation, Leiden University Fund and Innovation Fund Dutch Healthcare Providers; FH reports consultancy and lecture fees form ABBOTT, Saluda, Grunenthal and Pfizer; RB, EZ, LW, JH, IC and MF report no relevant conflict of interest. No funding was received for the ICON study group.

## Appendix A Supplementary data

Supplementary Figures and Tables

Study protocol

PubMed table

ICON group
