# Supplementary material for: A prospective open label 2–8 year extension of the randomised controlled ICON trial on the long-term efficacy and safety of occipital nerve stimulation in medically intractable chronic cluster headache
Source: eBioMedicine. 2023 Nov 25;98:104895. doi: 10.1016/j.ebiom.2023.104895 (PMC10755111; doi:10.1016/j.ebiom.2023.104895)
Supplement: Supplementary Figures and Tables [file mmc1.pdf]

Table of contents

Supplemental table 1	1
Supplemental table 2	1
Supplemental table 3	2
Supplemental figure 1	5
Supplemental figure 2	6
Supplemental figure 3	7
Supplemental figure 4	9
Appendix 1	10

Supplemental table 1 – (a) Pooled geometric mean weekly attack frequency with 95% CI after multiple imputations; (b) Pooled mean SF-36 scores with 95% CI after multiple imputations

**a**	**Baseline**	**48 weeks**	**1 year**	**2 years**	**3 years**	**4 years**	**5 years**
N with original data	**128**	**114**	**67**	**64**	**57**	**42**	**25**
**Weekly attack frequency**	16,2 *[14,4 - 18,3]*	3,8 *[2,7 - 5,4]*	4,2 *[2,8 - 6,3]*	5,1 *[3,5 - 7,6]*	4,4 *[3,1 - 6,2]*	3,8 *[2,7 - 5,2]*	4,1 *[3,0 - 5,5]*

**b**	**Baseline**	**48 weeks**	**1 year**	**2 years**	**3 years**	**4 years**	**5 years**
N with original data	**128**	**111**	**70**	**65**	**58**	**42**	**25**
**SF-36 PHS**	50,6 *[47,4 - 53,8]*	61,4 *[57,6 - 65,2]*	62,0 *[59,3 - 64,7]*	60,7 *[57,9 - 63,5]*	65,3 *[62,7 - 67,9]*	61,9 *[59,6 - 64,2]*	61,7 *[59,6 - 63,8]*
**SF-36 GH**	45,5 *[41,9 - 49,1]*	51,0 *[47,4 - 54,6]*	54,3 *[51,6 – 57,0]*	54,6 *[52,0 - 57,2]*	54,3 *[51,9 - 56,7]*	53,9 *[51,5 - 56,3]*	57,4 *[55,3 - 59,5]*
**SF-36 MHS**	47,1 *[43,0 - 51,2]*	60,1 *[55,8 - 64,4]*	64,7 *[61,9 - 67,5]*	63,0 *[60,1 - 65,9]*	67,4 *[64,9 - 69,9]*	64,6 *[62,2 - 67]*	70,4 *[68,3 - 72,5]*

Abbreviations: SF-36 = 36-Item Short-Form Health Survey; CI = Confidence interval; PHS = Physical health sumscore; GH = General health; MHS = Mental health sumscore

Supplemental table 2 – Binary logistic regression for odds for 50% attack reduction 2 years after implantation

	**Odds ratio [95% CI]**	**P value**
**Age**	1.02 [0.97 – 1.07]	0.428
**Sex**	0.85 [0.24 – 2.95]	0.795
**Number of years with CH**	1.07 [0.99 – 1.17]	0.096
**Number of autonomic symptoms**	0.91 [0.68 – 1.23]	0.550
**Attack frequency at baseline**	1.02 [0.98 – 1.07]	0.356
**Restlessness**	0.56 [0.10 – 3.03]	0.500

Supplemental table 3 – Adverse events from completion of the ICON trial (48 weeks after implantation + 10 days run-in) to last follow-up

	Events reported (n)	Patients with event (N, %)	Incidence rate (person-year^{-1}, [95% CI])
**Hardware-related that required surgery**			
Battery replacement	43	25 (40%)	0.13 [0.09 – 0.17]
Lead replacement	43	25 (28%)	0.13 [0.09 – 0.17]
Lead migration	26	19 (29%)	0.08 [0.05 – 0.11]
**Total**	112	48 (55%)	0.35 [0.28 – 0.41]
**Hardware-related that did not required surgery**			
Pain	11	8 (9%)	0.03 [0.01 – 0.05]
**Non-hardware related**			
Gastrointestinal	12	8 (9%)	
Orthopedic surgery	6	6 (7%)	
Chest pain	4	4 (5%)	
Inguinal hernia	4	2 (2%)	
Carcinoma	3	3 (3%)	
Temporarily loss of consciousness	3	3 (3%)	
Cardiac arrhythmia	2	2 (2%)	
Brain ischaemia	2	2 (2%)	
Laminectomy	2	2 (2%)	

Gynaecological	2	2 (2%)	
Benign tumour	2	2 (2%)	
Dental surgery	2	1 (1%)	
SPG blockade	2	1 (1%)	
Not specified	19	15 (17%)	
Other	14	14 (16%)	
**Total**	79	40 (45%)	0.24 [0.19 – 0.30]
**Hardware-related non-serious adverse event**			
Neck stiffness	194	51 (58%)	0.60 [0.52 – 0.68]
Local paraesthesia	136	41 (47%)	0.42 [0.35 – 0.49]
Unpleasant sensation	135	51 (58%)	0.42 [0.35 – 0.49]
Pain near the battery	116	28 (32%)	0.36 [0.29 – 0.42]
Inflammation near the battery	12	9 (10%)	0.04 [0.02 – 0.06]
**Total**	593	71 (81%)	1.83 [1.68 – 1.78]

Supplemental figure 1 – Kaplan Meier curve representing time to first attainment of ≥50% (a) and time to first attainment of ≥30% (b) attack reduction with 95% confidence interval. All participants that did not achieve a response 5 years after the ICON trial were censored.

(a)

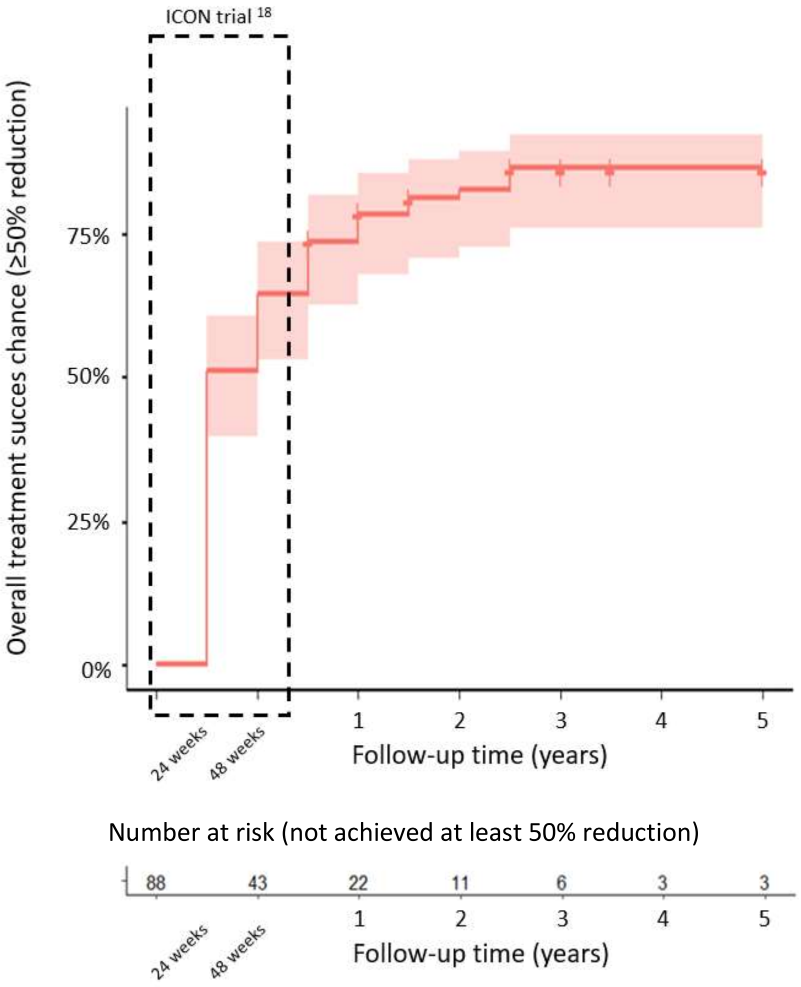

(b)

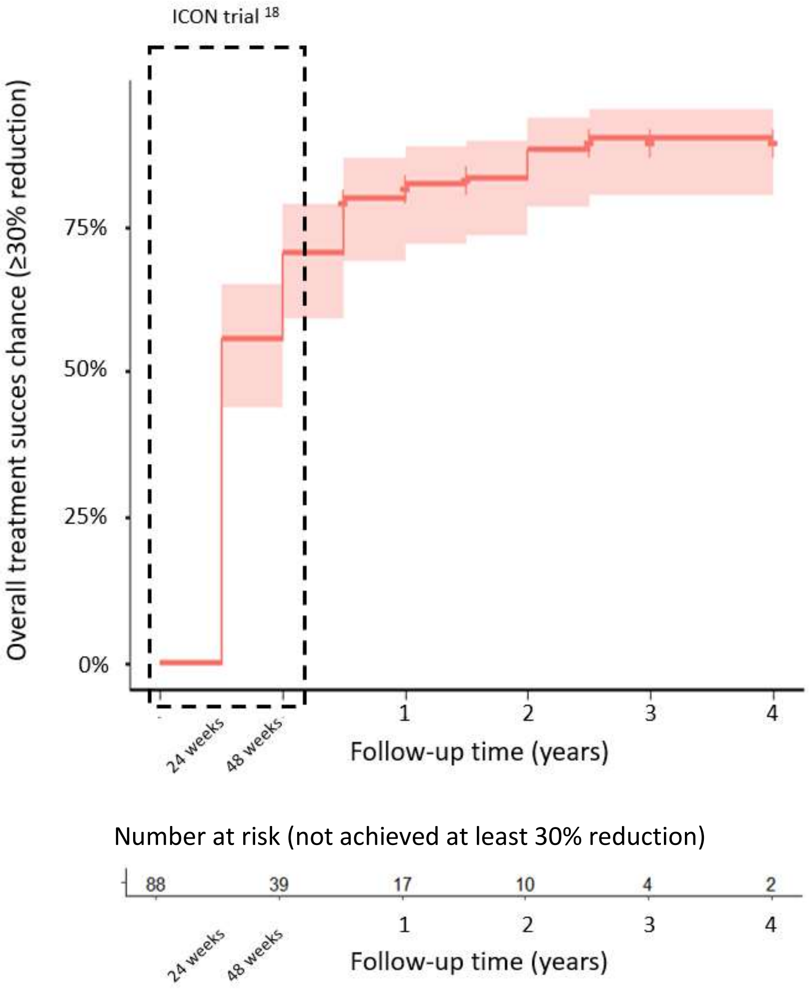

Supplemental figure 2 – Heat maps depicting each individual participants' subjective response to ONS until 8.5 years after ICON study completion. Individual follow-up duration is dependent on enrolment date. Explantation (n=8), device turned off due to no effect (n=3) and no effect (n=2) have been marked as 'dropout' (n=13). End of follow-up due to study ending (n=57), lost to follow-up (n=6), personal reasons (n=6), death due to other disease (n=3) or attack freedom (n=3) have been marked as 'censored' (n=75)

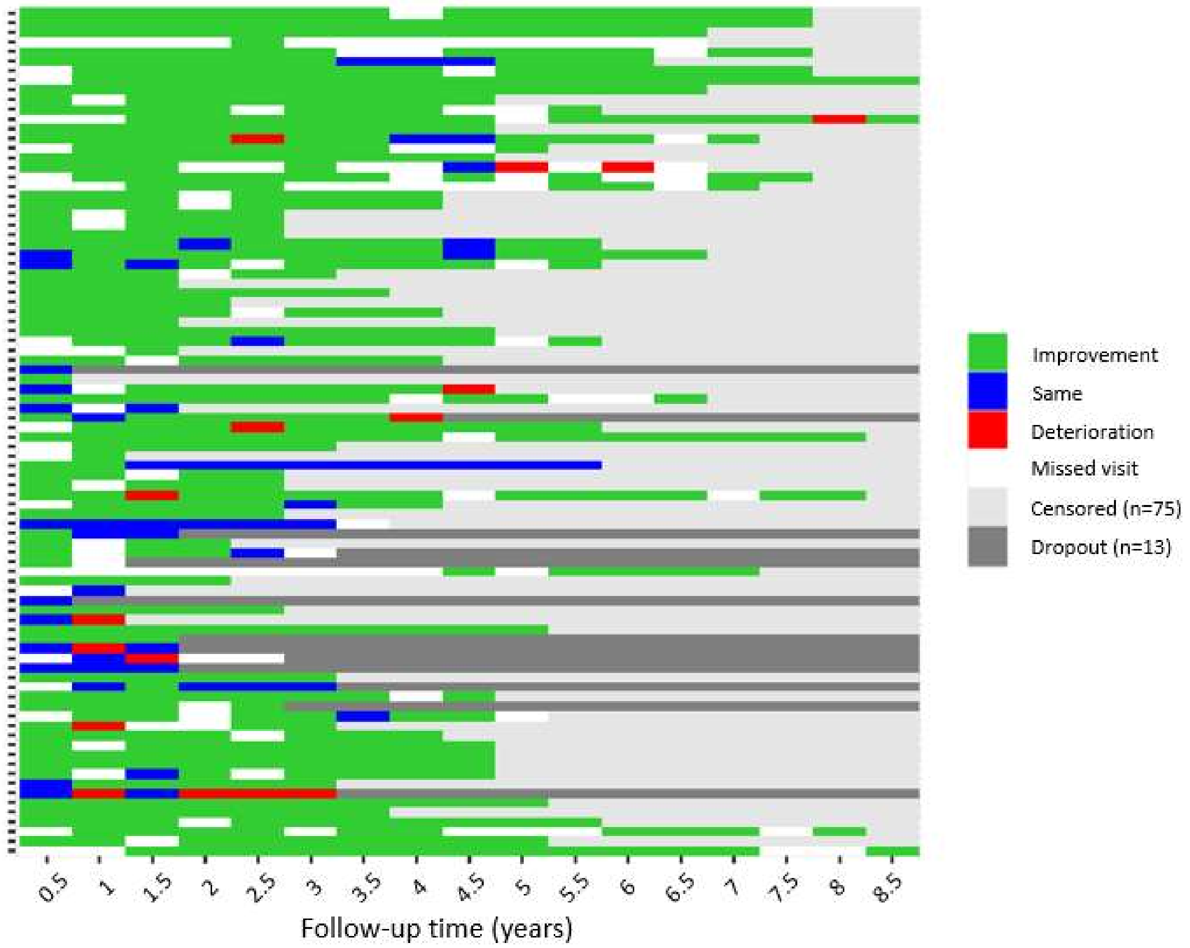

Supplemental figure 3 – Comparison between imputed data and complete case analysis for: (a) Mean weekly attack frequency; (b) Mental health sumscore; (c) Physical health sumscore and (d) General health. All with 95% CI.

a

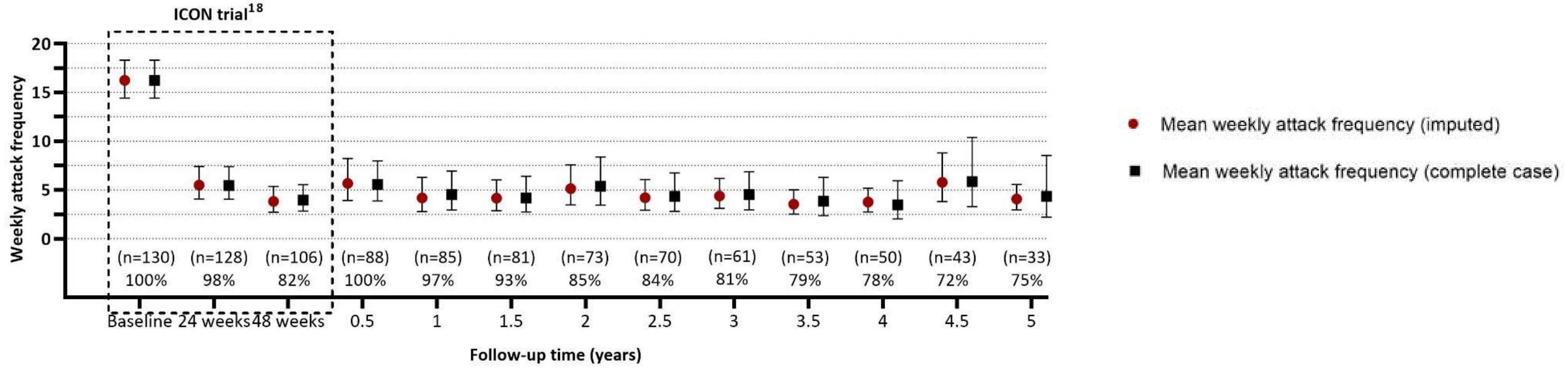

b

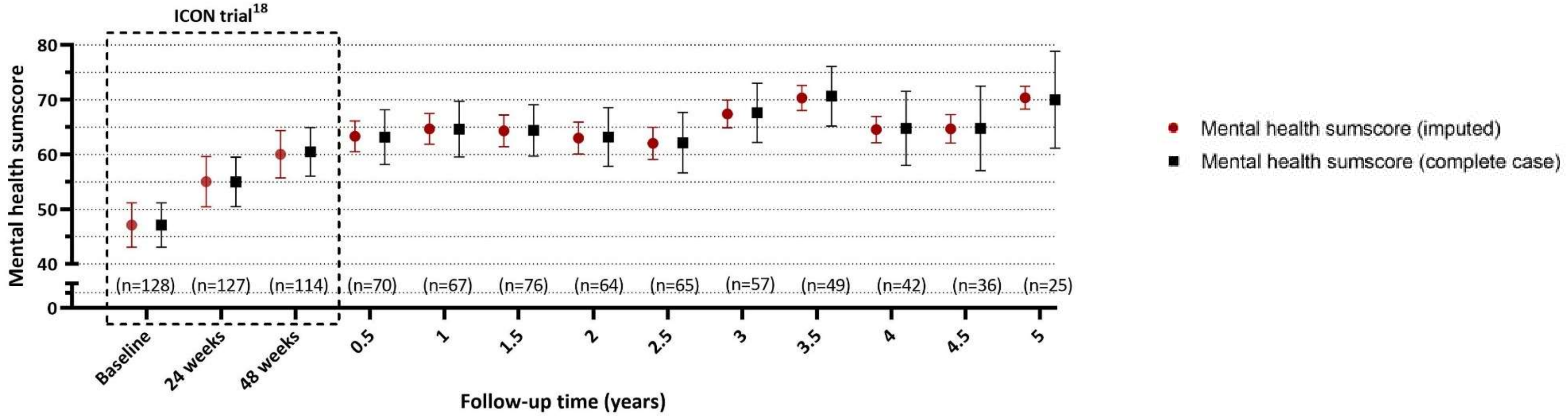

c

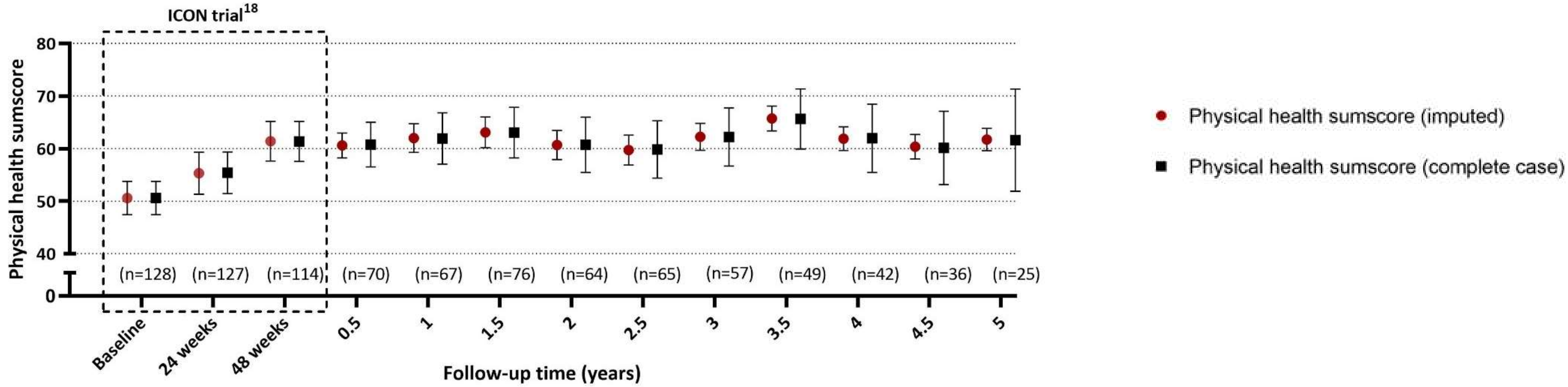

ICON trial18
Physical health sumscore
80
70
60
50
40
0
(n=128) (n=127) (n=114) (n=70) (n=67) (n=76) (n=64) (n=65) (n=57) (n=49) (n=42) (n=36) (n=25)
Baseline
24 weeks
48 weeks
0.5
1
1.5
2
2.5
3
3.5
4
4.5
5
Follow-up time (years)
Physical health sumscore (imputed)
Physical health sumscore (complete case)

d

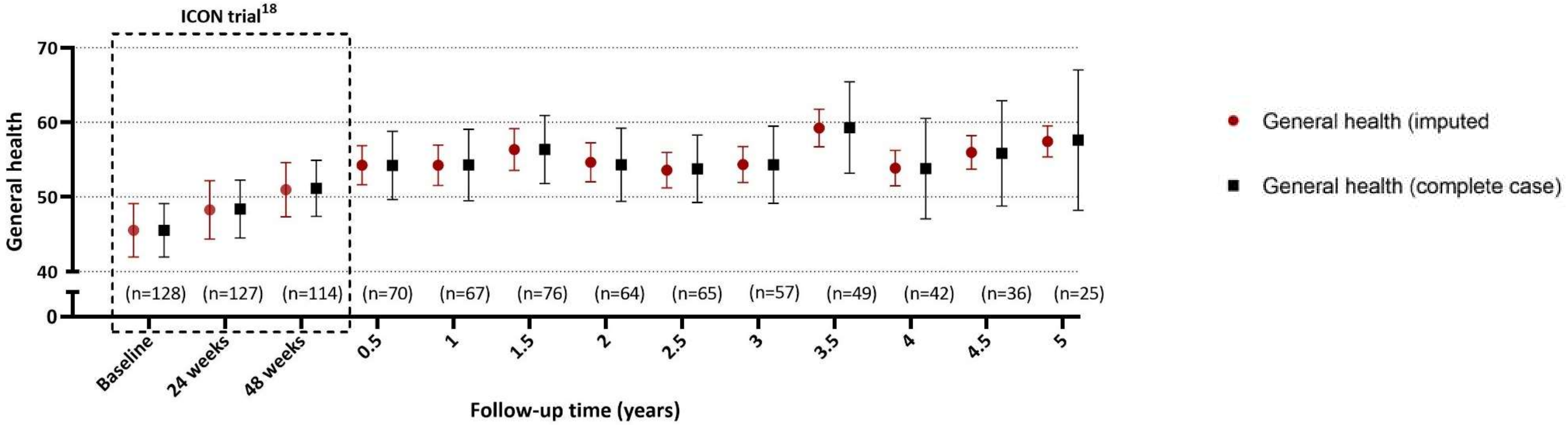

ICON trial18
General health
70
60
50
40
0
(n=128) (n=127) (n=114) (n=70) (n=67) (n=76) (n=64) (n=65) (n=57) (n=49) (n=42) (n=36) (n=25)
Baseline
24 weeks
48 weeks
0.5
1
1.5
2
2.5
3
3.5
4
4.5
5
Follow-up time (years)
General health (imputed
General health (complete case)

Supplemental figure 4 – Sensitivity analyses in which we increased and decreased all previously imputed attack frequencies in the participants that were missing data for other reasons than study termination by 50%.

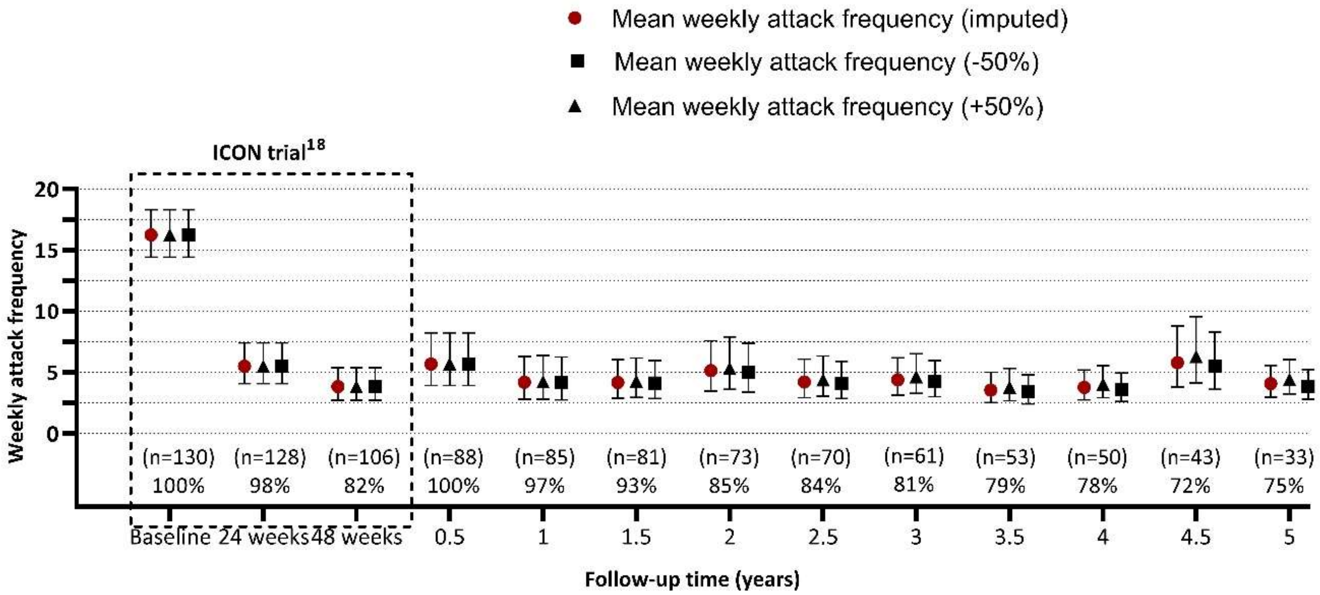

Appendix 1

ICON study group

Investigators are listed by center

1. Leiden University Medical Center - M.D. Ferrari (Chair), L.A. Wilbrink, I. F. de Coo, P.G.G. Doesborg, E.C. Bartels, E.W. van Zwet
2. Erasmus Medical Center - F.J.P.M. Huygen (Vice Chair)
3. Canisius Wilhelmina Hospital - W. Mulleners, E. Kurt
4. Radboud Medical Center - R.T.M. van Dongen
5. Zuyderland Hospital - O.P. Teernstra, P.J.J. Koehler, G.H. Spincemaille
6. Diakonessenhuis Zeist - F. Wille
7. Alrijne Hospital - K. Burger, J. Haan
8. Boerhaave Medical Center - E.G.M. Couturier
9. Rijnstate Hospital Arnhem - J.W. Kallewaard
10. University of Twente – Peter H. Veltink
11. Medtronic BV - R. Buschman
