# Supplementary material for: A prospective open label 2–8 year extension of the randomised controlled ICON trial on the long-term efficacy and safety of occipital nerve stimulation in medically intractable chronic cluster headache
Source: eBioMedicine. 2023 Nov 25;98:104895. doi: 10.1016/j.ebiom.2023.104895 (PMC10755111; doi:10.1016/j.ebiom.2023.104895)
Supplement: Study protocol [file mmc2.pdf]

Long term follow-up after occipital nerve stimulation

Addendum 3 CME protocol P10.016

Version: 1.1

Date: 03-08-2016

Clinical investigators:

P.G.G. Doesborg, MD

Principle investigator:

Prof. M.D. Ferrari, MD, PhD

Co-investigators/ Supervisors:

J. Haan, MD, PhD

Department of Neurology, LUMC, Leiden, The Netherlands

Further involved:

Department of Neurology, LUMC, Leiden, The Netherlands

L.A, Wilbrink, MD (previous investigator)

I.F. de Coo, MD (previous investigator)

Department of Neurosurgery, MUMC+, Maastricht, The Netherlands

Prof. Veerle Visser-Vandewalle, MD, PhD

Onno Teernstra, MD, PhD

Geert Spincemaille, MD, PhD

Department of Anesthesiology, Erasmus MC, Rotterdam, The Netherlands

Prof Frank J.P.M. Huygen, MD, PhD

Department of Biomedical signals and systems, University of Twente, Enschede, The Netherlands

Prof. Peter H. Veltink, PhD

Department of Neurology, Canisius-Wilhelmina Hospital, Nijmegen, The Netherlands

Wim M. Mulleners, MD, PhD

Department of Medical Statistics & BioInformatics, LUMC, Leiden, The Netherlands

Prof. Ronald Brand, PhD

Erik van Zwet, PhD

Faculty of Health, Medicine and Life Sciences, Capaciteitsgroep Beleid & Organisatie van de zorg –BEOZ, MUMC+, Maastricht, The Netherlands

Silvia Evers, PhD

Funding: Medtronic

Unrestricted grant

Non voting representative Department of Benelux and Nordic Region, Medtronic, Heerlen

Rik P.J. Buschman, PhD

TABLE OF CONTENTS

ABSTRACT 4
1. Background 5
2. Study rationale 5
3. Study design 6
4. Methods 6
Subjects 6
Endpoints 6
Inclusion and exclusion criteria 7
Measurements 7
Informed consent 8
Storage of data 8
Statistics/ Sample sizes needed 8
Economic evaluation 8
3. Unexpected clinical findings 9
4. Participation payment 9
5. Independent physician 9
Appendices 10
- Appendix A: Long term follow up questionnaire 10
- Appendix B: Economic evaluation questionnaire 10
- Appendix C: Information letter and informed consent 10

ABSTRACT

Objective: To evaluate long term outcome of occipital nerve stimulation (ONS) in terms of (cost-) effectiveness, mean attack frequency (MAF) and adverse events in patients suffering from medically intractable chronic cluster headache (MICCH).

Background: Patients suffering from MICCH are included in the ICON study and have been randomized to high or low stimulation for six months. After this blinded period of six months, patients receive supposed optimal stimulation in an open phase for six months. Thus, the total follow-up of the ICON study is one year.
A recent study of Magis et al. showed data on long term follow up of ONS in 15 patients suffering from MICCH with a mean follow up of 36.82 months. At least 11 patients of the 14 patients showed at least 90% reduction in headache attacks and 9 of them become pain-free for prolonged periods. (1) Burns et al. showed improvement in 6 out of 8 patients after a median follow up of 20 months. (2) There are, however, no large, prospective long term follow-up studies on (cost-) effectiveness, quality of life and adverse events in MICCH patients treated with ONS.
In this study patients participating in the ICON study (n=144) will be asked to participate in a long term follow up program, which consists of a questionnaire every six months for three years or more and an interview by phone at 12, 18 and 24 months ONS treatment. These data will potentially give us insight in the long term effects of ONS in MICCH patients.

Methods: Patients who participated in the ICON study will be contacted every six months by email or phone to fill in a web-based questionnaire. The questionnaire will include questions on attack frequency, quality of life, and possible adverse events. Additional, the patients will be interviewed at 12, 18 and 24 months by phone for a long term economic evaluation.

1. Background

Some small, open, non-randomized studies have shown improvement in headache attack frequency with occipital nerve stimulation (ONS) in patients with medically intractable chronic cluster headache (MICCH). Burns et al. described 14 patients suffering from MICCH who were treated with occipital nerve stimulation ONS in an open retrospective study. Ten patients improved; three improved by 90% or more, 3 by 40%-90% and 4 by 20-30%. (2;3) In another prospective open ONS study on MICCH patients Magis et al. showed a reduction in attack frequency of 79.9%. (20) A total of 91 patients with MICCH and ONS are described in the literature and 67% of them reported an improvement of headache frequency of at least 50%. (4) No serious complications of ONS were described.
Recently, Magis et all. showed data on long term follow up of ONS in 15 MICCH patients with a mean follow up of 36.82 months. 11 out of 14 patients showed at least 90% reduction in frequency of headache attacks, 9 of them became pain-free for prolonged periods. The most common adverse event was battery depletion due to the use of high current intensities in 9 patients. 5 patients underwent a surgery to remove the device, mostly because of an inflammation nearby the site of implantation. (1) Burns et al. showed an improvement of 6 out of 8 MICCH patients at a median follow up of 20 months. 2 patients had a reduction of 90% in headache attacks. (2)
To our best knowledge, there are at the moment no large, prospective long term follow-up studies on (cost-) effectiveness, quality of life and adverse events in MICCH patients treated with ONS.

2. Study rationale

Long term effects of ONS are promising, but only assessed in small studies. (1;2;5) Therefore, a large prospective follow up study can be of great value to assess the effect on the mean headache attack frequency, cost effectiveness, quality of life and possible adverse events.

3. Study design

In this follow up study patients participating in the ICON study will be asked to participate in a long term follow up program. The ICON study is a prospective, randomized, double blind, parallel group multi-centre international clinical study to compare the reduction in attack frequency from baseline and after occipital nerve stimulation (ONS) in MICCH patients between two different stimulation conditions: high (100%) and low (30%) stimulation. A total of 144 patients will be included. The follow up of the ICON study is 1 year after implantation. In the long term follow up the mean headache attack frequency, (cost) effectiveness, quality of life and adverse events will be measured. The program will consist of a digital questionnaire every six months for at least three years. In addition, at 12, 18 and 24 months an economic evaluation will be carried out by means of a telephone interview. These data will give us insight in long term effects of ONS in MICCH patients.

4. Methods

Subjects

All patients participating in the ICON study will be asked to take part in this study.

Endpoints

Primary endpoint:

The primary endpoint is the mean headache attack frequency at baseline compared to the mean headache attack frequency (MAF) at 2 years after ONS surgery.

Secondary endpoints:

- MAF: We will ask the patient every half year the mean attack frequency of the past 4 weeks. We will compare the baseline MAF with the MAF at 12 months and 18 months.
- Patient satisfaction: We will ask the patient whether he/she would recommend the treatment to another patient. This will be expressed in a 5 point (Likert) scale: strongly disagree, disagree, neither agree nor disagree, agree, strongly agree. This evaluation will take place every half year.

- Adverse events: All and treatment-related adverse events will be documented by the investigators at 18, 24, 30 etc months follow up and if necessary more frequent.
- Use of stimulator: We will ask the patients how many hours per day the stimulator is switched on. This evaluation will take place every half year.
- Quality of life: We will ask the patients to fill in the SF-36 questionnaire every half year.
- Economic evaluation: We will ask the patient to fill in an economic questionnaire to evaluate the use of resources at 2 year follow-up.

Inclusion and exclusion criteria

The inclusion and exclusion criteria are according to the ICON study. All patients included in the ICON study will be invited.

Measurements

Patients will be invited to fill in a (electronic) questionnaire at 18, 24, 30 etc months follow up. Patients will be approached at 12, 18 and 24 months for an additional economic evaluation by means of an interview by telephone.

	T=1	T=2	T=3	T=4	T=5	T=…
	12 months after surgery	18 months after surgery	24 months after surgery	30 months after surgery	36 months after surgery	…
Visit window	+ 8 weeks	+/- 6 weeks	+/- 6 weeks	+/- 6 weeks	+/- 6 weeks	+/- 6 weeks
SF-36		X	X	X	X	X
MAF		X	X	X	X	X
Economic evaluation	X	X	X			
Adverse events		X	X	X	X	X

Informed consent

Patients are recruited at the end of or shortly after finishing the ICON-trial. All patients will sign an informed consent for this long term follow up study. At any time patients can withdraw from this study without any explanation.

Storage of data

Data from the follow up study will be coded and stored in the ProMISe database in accordance with current legislation. This database has already been reported at the College Bescherming Persoonsgegevens (CBP). Data that can be used to identify persons will only be accessible for members of the ICON research team. Persons concerned from the Department of Medical Statistics have been enjoined secrecy, as was stated in the above mentioned supplement. This secrecy includes all data in ProMISe.

Statistics/ Sample sizes needed

Because this study will be in embedded in the ICON study no separate power calculations will be performed for the follow up study.

To analyse the mean headache attack frequency between baseline and 2 years ONS treatment we will perform a regression analysis if the data are normally distributed. To analyse quality of life, as assessed with the standardized SF-36, we will perform a regression analysis if the data is normally distributed. Adverse events will be analysed using descriptive statistics. Further analyses will be performed in concordance with views of the department of Medical Statistics.

Economic evaluation

The objective of the economic evaluation part of this study is to compare the costs, effects and utilities from a societal perspective between baseline and 24 months of follow up. This economic evaluation will involve a combination of a cost-effectiveness analysis and a cost-utility analysis.

In a cost-effectiveness analysis effects are presented in clinical outcomes (in our study mean frequency of CH attacks during the last 4 weeks of the baseline, after 6 months and 2 years ONS).The primary outcome measure for the cost-utility analysis

will be Quality Adjusted Life Years (QALYs), based on the SF-36 utility scores. See for calculations details in the ICON study protocol. (6-8)

No power calculations will be performed for the economic evaluation study as it is embedded in a follow up study of an RCT. For the analyses we will use SPSS statistical software.The analyses will be performed in concordance with views of the department of Medical Statistics.

3. Unexpected clinical findings

Not applicable in this study.

4. Participation payment

Study subjects will not receive payment for their participation.

5. Independent physician

Prof. Dr. J.G. van Dijk of the Neurology department has agreed to act as an independent physician (telephone number: +3171-5262895).

References

(1) Magis D, Gerardy PY, Remacle JM, Schoenen J. Sustained effectiveness of occipital nerve stimulation in drug-resistant chronic cluster headache. Headache 2011 Sep;51(8):1191-201.

(2) Burns B, Watkins L, Goadsby PJ. Treatment of medically intractable cluster headache by occipital nerve stimulation: long-term follow-up of eight patients. Lancet 2007 Mar 31;369(9567):1099-106.

(3) Burns B, Watkins L, Goadsby PJ. Treatment of intractable chronic cluster headache by occipital nerve stimulation in 14 patients. Neurology 2009 Jan 27;72(4):341-5.

(4) Magis D, Schoenen J. Advances and challenges in neurostimulation for headaches. Lancet Neurol 2012 Aug;11(8):708-19.

(5) Mueller OM, Gaul C, Katsarava Z, Diener HC, Sure U, Gasser T. Occipital nerve stimulation for the treatment of chronic cluster headache - lessons learned from 18 months experience. Cent Eur Neurosurg 2011 May;72(2):84-9.

(6) Brazier J, Roberts J, Deverill M. The estimation of a preference-based measure of health from the SF-36. J Health Econ 2002 Mar;21(2):271-92.

(7) Brazier JE, Harper R, Jones NM, O'Cathain A, Thomas KJ, Usherwood T, et al. Validating the SF-36 health survey questionnaire: new outcome measure for primary care. BMJ 1992 Jul 18;305(6846):160-4.

(8) van der Zee KI, Sanderman R. Het meten van de algemene gezondheidstoestand met de Rand-36: Een handleiding. Noordelijk Centrum voor Gezondheidsvraagstukken. RUG; 1993.

Appendices

- Appendix A: Long term follow up questionnaire
- Appendix B: Economic evaluation questionnaire
- Appendix C: Information letter and informed consent

Appendix A: long term follow up questionnaire

Lange termijn (> 1 year) follow-up ICON study

Promise ID
Naam
Geboorte datum

1. Hoeveel uur per dag staat de stimulatie aan?

- 24/7 (altijd)
- Anders, namelijk

2. Hoeveel aanvallen van clusterhoofdpijn heeft u gemiddeld per week gehad de afgelopen tijd? (deze vraag gaat over de afgelopen 4 weken.)

-

3. Als u de clusterhoofdpijn van nu vergelijkt met voor de stimulatie. Ervaart u dan een verbetering?

- Ja, verbetering: … %
- Nee, het is hetzelfde gebleven
- Nee, verslechtering … %

4. Zou u deze behandeling aanraden aan een andere patiënt met soortgelijke hoofdpijnklachten?

0 Sterk mee eens
0 Mee eens
0 Niet mee eens/ niet mee oneens
0 Oneens
0 Sterk mee oneens

5. Heeft u het **afgelopen half jaar** bijwerkingen van de batterij of stimulator bemerkt? (Het is mogelijk om meer dan één antwoord te kiezen)

- Ja,

    - De batterij was leeg
    - Stijfheid in de nek
    - Onplezierige gewaarwording van tintelingen
    - Verplaatsing van een elektrode
    - Pijn bij de batterij
    - Ontsteking bij de batterij of elektrode
    - Anders namelijk ……….
- Nee

6. Bent u voor (een van deze) bijwerkingen opgenomen geweest in het ziekenhuis?
    - Ja
    - Nee

   Indien ja, hoevaak…

7. Bent u voor deze bijwerking geopereerd (bijvoorbeeld een nieuwe batterij of elektrode)
    - Ja
    - Nee

   Indien ja, hoevaak….

SF-36

TOELICHTING: Deze vragenlijst gaat over uw standpunten ten aanzien van uw gezondheid. Met behulp van deze gegevens kan worden bijgehouden hoe u zich voelt en hoe goed u in staat bent uw gebruikelijke bezigheiden uit te voeren.

Beantwoord elke vraag door het juiste hokje aan te kruisen Als u niet zeker weet hoe u een vraag moet beantwoorden, geef dan het best mogelijke antwoord.

1. **Hoe zou u over algemeen uw gezondheid noemen? (kruis het hokje aan)**

Uitstekend	1
Zeer goed	2
Goed	3
Matig	4
Slecht	5

2. **Hoe beoordeelt u <u>nu</u> uw gezondheid over het algemeen, <u>vergeleken met een jaar geleden</u>? (kruis het hokje aan)**

Veel beter nu dan een jaar geleden	1
Wat beter nu dan een jaar geleden	2
Ongeveer hetzelfde nu als een jaar geleden	3
Wat slechter nu dan een jaar geleden	4
Veel slechter nu dan een jaar geleden	5

3. **De volgende vragen gaan over bezigheden die u misschien doet op een doorsnee dag. Wordt u door uw <u>gezondheid op dit moment beperkt</u> bij deze bezigheden? Zo ja, welke mate? (kruis het hokje aan)**

	**BEZIGHEDEN**	**Ja, ernstig beperkt**	**Ja, een beetje beperkt**	**Nee, helemaal niet beperkt**
A	**Forse inspanning**, zoals hardlopen, tillen van zware voorwerpen, een veeleisende sport beoefenen	1	2	3
B	**Matige inspanning**, zoals een tafel verplaatsen, stofzuigen, zwemmen of fietsen	1	2	3
C	Boodschappen tillen of dragen	1	2	3
D	**Een paar** trappen oplopen	1	2	3
E	**Één** trap oplopen	1	2	3
F	Bukken, knielen of hurken	1	2	3
G	**Meer dan een kilometer** lopen	1	2	3
H	**Een paar honderd meter** lopen	1	2	3
I	Ongeveer **honderd meter** lopen	1	2	3
J	Uzelf wassen of aankleden	1	2	3

4. **Heeft u in de <u>afgelopen 4 weken</u> één van de volgende problemen bij uw werk of andere dagelijkse bezigheden gehad, <u>ten gevolge van uw lichamelijke gezondheid</u>? (kruis het hokje aan)**

		**Ja**	**Nee**
A	U besteedde **minder tijd** aan het werk of andere bezigheden	1	2
B	U heeft **minder bereikt** dan u zou willen	1	2
C	U was beperkt in het **soort** werk of andere bezigheden	1	2
D	U had **moeite** om uw werk of andere bezigheden uit te voeren (het kostte u bij voorbeeld inspanning)	1	2

5. Heeft u in de afgelopen 4 weken, één van de volgende problemen ondervonden bij uw werk of andere dagelijks bezigheden ten gevolge van emotionele problemen (zoals depressieve of angstige gevoelens)? (kruis het hokje aan)

		Ja	Nee
A	U besteedde **minder tijd** aan werk of andere bezigheden	1	2
B	U heeft **minder bereikt** dan zou u willen	1	2
C	U deed uw werk of andere bezigheiden niet zo **zorgvuldig** als gewoonlijk	1	2

6. In hoeverre hebben uw lichamelijke gezondheid of emotionele problemen u gedurende de afgelopen 4 weken gehinderd in uw normale omgang met familie, vrienden of buren, of bij activiteiten in groepsverband? (kruis het hokje aan)

Helemaal niet	1
Enigszins	2
Nogal	3
Veel	4
Heel erg veel	5

7. Hoeveel lichamelijke pijn heeft u de afgelopen 4 weken gehad? (kruis het hokje aan)

Geen	1
Heel licht	2
Licht	3
Nogal	4
Ernstig	5

Zeer ernstig	6

8. In welke mate bent u Afgelopen 4 weken door pijn gehinderd in uw normale werk (zowel werk buitenhuis als huishoudelijk werk) ? (kruis het hokje aan)

Helemaal niet	1
Een klein beetje	2
Nogal	3
Veel	4
Heel erg veel	5

9. Deze vragen gaan over hoe u zich voelt en hoe het met u ging in de afgelopen 4 weken. Wilt u a.u.b. bij elke vraag het antwoord geven dat het best benadert hoe u zich voelde. (kruis het hokje aan)

	**Hoe vaak gedurende de afgelopen 4 weken ..**	**altijd**	**meestal**	**vaak**	**soms**	**zelden**	**nooit**
A	..voelde u zich levenslustig?	1	2	3	4	5	6
B	..was u erg zenuwachtig?	1	2	3	4	5	6
C	..zat u zo in de put dat niets u kon opvrolijken?	1	2	3	4	5	6
D	..voelde u zich rustig en tevreden?	1	2	3	4	5	6
E	..had u veel energie?	1	2	3	4	5	6
F	..voelde u zich somber en neerslachtig?	1	2	3	4	5	6
G	..voelde u zich uitgeput?	1	2	3	4	5	6
H	..was u een gelukkig mens?	1	2	3	4	5	6
I	..,voelde u zich moe?	1	2	3	4	5	6

10. Hoe vaak hebben uw lichamelijke gezondheid of emotionele problemen u gedurende de afgelopen 4 weken gehinderd bij uw sociale activiteiten (zoals vrienden of familie bezoeken, etc.)? (kruis het hokje aan)

Altijd	1
Meestal	2
Soms	3
Zelden	4
Nooit	5

11. Hoe JUIST of ONJUIST is elk van de volgende uitspraken voor u? (kruis het hokje aan)

		**volkomen juist**	**grotendeels juist**	**weet ik niet**	**grotendeels onjuist**	**volkomen onjuist**
A	Ik lijk wat gemakkelijker ziek te worden dan andere mensen die ik ken	1	2	3	4	5
B	Ik ben even gezond als andere mensen die ik ken	1	2	3	4	5
C	Ik verwacht dat mijn gezondheid achteruit zal gaan	1	2	3	4	5
D	Mijn gezondheid is uitstekend	1	2	3	4	5

Bedankt voor het invullen

Appendix B: Economic evaluation questionnaire

Vragenlijst kostenonderzoek

Lange termijn follow-up ICON studie

Naam interviewer:

Naam patiënt

Geslacht

Geboortedatum

Datum interview

Module A Algemeen

A1. Wat is de hoogste opleiding die u heeft afgerond?

- Lagere school
- Lager beroepsonderwijs (Huishoudschool, LTS, LEAO, VBO enz.)
- VMBO, MAVO, (M)ULO, MMS, drie jaar HBS
- HAVO, VWO, HBS
- Middelbaar beroepsonderwijs (MTS, MEAO, BOL, NAS enz.)
- Hoger beroepsonderwijs (HTS, HEAO, Sociale Academie, HBO enz.)
- Universiteit

Module B Beroep, inkomen en werksituatie

B1. Heeft u op dit moment betaald werk of zit u in de ziektewet?

☐ja

☐nee, **ga verder naar module F**

B2. Voor hoeveel uur per week heeft u een aanstelling? ……. uren per week

B3. Over hoeveel dagen zijn deze uren verdeeld? …….. dagen

B4. Wat is uw beroep? …………………………………………………………………….

B5. Wat is uw eigen netto inkomen uit betaald werk?

Het gaat het om het bedrag dat u 'schoon' (netto) in uw handen krijgt. Het gaat alleen om uw eigen inkomen, dus zonder dat van uw eventuele partner.

Het eigen netto inkomen uit betaald werk is *(maar 1 regel invullen)*

………..………………………….. Euro per week

………..………………………….. Euro per 4 weken

………………………………….. Euro per maand

…….. ………………………….. Euro per jaar

Dat weet ik niet, dat wil ik niet zeggen.

Wij zouden u graag een aantal vragen willen stellen over uw werksituatie.

**Werksituatie**	Nooit	Soms	Vaak	Altijd
B6. Kunt u uw werk zelf indelen?	☐	☐	☐	☐
B7. Is uw werk uit te stellen?	☐	☐	☐	☐
B8. Leert u nieuwe dingen in uw werk?	☐	☐	☐	☐
B9. Heeft u mogelijkheden om uw eigen capaciteiten verder te ontwikkelen?	☐	☐	☐	☐
B10. Hoe vaak heeft u binnen uw werk te maken met ingewikkelde problemen?	☐	☐	☐	☐

B11. Hoeveel collega's zijn er die hetzelfde of vergelijkbaar werk doen?
……… collega's met hetzelfde werk

B12. Kunt u op onderstaande schaal aankruisen hoe uw relatie is met uw collega's in uw huidige functie? U kunt dit aangeven door een getal van 0 tot 10 te kiezen, waarbij 0 "heel erg slecht" en 10 "heel erg goed" betekent

heel erg slecht 0 1 2 3 4 5 6 7 8 9 10 heel erg goed

B13. Verricht u onregelmatige- of ploegendiensten?

☐ ja

☐ nee

B14. Geeft u leiding aan personeel?

☐ ja

 nee

B15. Hoeveel werknemers heeft het bedrijf waar u werkt?

 < 50

 50-200

 > 200

B16. In welke sector is dit bedrijf werkzaam?

 industrie

 bouwnijverheid

 gezondheid en welzijn

 publieke sector (politie, gemeente)

 onderwijs

 commerciële dienstverlening (bank, winkel, horeca, garage)

 anders, namelijk...

Module C Verzuim

C1. Heeft u zich gedurende de **afgelopen zes maanden** ziek gemeld voor uw werk?

- ja
- nee → **ga naar module E**

C2. Verzuimt u op dit moment?

- ja
- nee, wat is de datum van de laatste verzuimdag?

……………………**dag maand Jaar**

C3. Hoeveel **werk**dagen (in totaal) heeft u de **afgelopen zes maanden** verzuimd?

…. Werkdagen

C4. Hoe vaak heeft u de afgelopen 6 maanden verzuimd?

…. periodes

Module D Compensatiemechanismen bij verzuim

D1. Hoeveel **werk**dagen heeft u verzuimd tijdens **de kortste** verzuimperiode in de
afgelopen zes maanden?

…. Werkdagen

D2. Is uw werk overgenomen dan wel ingehaald, tijdens of na uw **kortste** verzuimperiode?

- □ collega's/ leidinggevende namen het werk over in normale uren
- □ collega's/ leidinggevende namen het werk over door overwerk
- extra krachten namen het werk over
- u heeft het werk zelf later tijdens normale uren ingehaald
- u heeft het werk zelf later tijdens overuren ingehaald
- het werk is niet overgenomen of ingehaald
- u weet niet hoe het werk is overgenomen

D3. Heeft u meerdere periodes verzuimd in de afgelopen 6 maanden?

- ja
- nee → **ga naar module E**

D4. Hoeveel **werk**dagen heeft u verzuimd tijdens **de langste** verzuimperiode in de
afgelopen zes maanden?
…. werkdagen

D5. Is uw werk overgenomen dan wel ingehaald, tijdens of na uw **langste** verzuimperiode?
- collega's/leidinggevende namen het werk over in normale uren
- collega's/leidinggevende namen het werk over door overwerk
- extra krachten namen het werk over
- u heeft het werk zelf later tijdens normale uren ingehaald
- u heeft het werk zelf later tijdens overuren ingehaald
- het werk is niet overgenomen of ingehaald
- u weet niet hoe het werk is overgenomen

Module E Productiviteitskosten tijdens werk

E1. Kunt u aangeven op een schaal van 0 tot 10 **hoeveel werk** u **tijdens uw laatste**
werkdag hebt gedaan in uw normale werktijd ten opzichte van een normale werkdag.
Een 0 betekent dat u niets kon doen en een 10 dat u evenveel als normaal kon doen.

Cijfer:

E2. Kunt u aangeven op een schaal van 0 tot 10 hoe de **kwaliteit** was van het werk dat u **tijdens uw laatste werkdag** hebt gedaan ten opzichte van normaal. Een 0 betekent
dat uw werk van zeer slechte kwaliteit was en een 10 dat u dezelfde kwaliteit heeft

geleverd als normaal.

Cijfer:

E3. Heeft u bij vraag E1 of E2 een cijfer 10 gegeven?

nee, vanwege:

- clusterhoofdpijn klachten
- andere gezondheidsproblemen
- problemen met werk (geen materiaal, machine kapot, etc.)

□anders, namelijk………………

- ja

Module F Zorgvraag

F1 *Hoe vaak heeft u gedurende de afgelopen 6 maanden, na aanleiding van uw clusterhoofdpijn, contact gehad met uw huisarts (indien contact gehad met vervanger ipv eigen huisarts dit contact ook meetellen).*
Indien nodig aantal keren schatten

- 01 geen enkele keer
- 02 ..... keer
- 09 weet niet/geen antwoord

Op soortgelijke wijze ga ik nu vragen stellen over de contacten met specialisten. (Specialistenhulp tijdens de ziekenhuisopname moet u niet meetellen, maar wel poliklinische behandeling, ook eerste hulp en foto's maken).

F2 *Hoe vaak heeft u de afgelopen 6 maanden contact gehad met een specialist?*
Indien nodig aantal keren schatten.

- 01 geen enkele keer
- 02 ..... keer
- 09 weet niet/geen antwoord

Nu volgen enkele vragen over het ziekenhuis

F3 *Heeft u de afgelopen 6 maanden wel eens in een ziekenhuis of kliniek gelegen?*
En zo ja, hoe vaak?

- 01 geen enkele keer, ga door naar F5
- 02 ..... keer
- 09 weet niet/geen antwoord

F4 *Hoeveel nachten heeft u de afgelopen 6 maanden in het ziekenhuis doorgebracht?*

- 01 geen enkele nacht
- 02 ..... nachten
- 09 weet niet/geen antwoord

F5 *Heeft u voor u zelf de afgelopen 6 maanden, gebruik gemaakt van een van de volgende gezondheidsvoorzieningen? Vervolgens willen ook graag weten hoe vaak u gebruik heeft gemaakt van deze voorziening.*

☐ fysiotherapie (buiten ziekenhuisopname) ..... keer
☐ oefentherapie (cesar) ..... keer
☐ chiropractor ..... keer
☐ biofeedback ..... keer
☐ mensendieck ..... keer
☐ ergotherapie ..... keer
☐ activiteitentherapie ..... keer
☐ maatschappelijk werk ..... keer
☐ acupunctuur ..... keer
☐ yoga ..... keer

 diëtist ..... keer

 massage ..... keer

 andere genezers, ... keer

……………………………. ..... keer

……………………………………. ..... keer

…………………………………… ..... keer

F6 *Heeft u de afgelopen 6 maanden wel eens gebruik gemaakt van psychiatrische/psychologische zorg (poliklinisch bezoek psycholoog, poliklinisch bezoek psychiater, GGZ)*
En zo ja, hoe vaak?

 Ja: … keer

 Nee

F7 *Heeft u de afgelopen 6 maanden wel eens gebruik gemaakt van thuiszorg, betaald of onbetaald (=mantelzorg=hulp van familie en/of bekenden)?*
En zo ja, hoeveel uur betaald en/of onbetaald?

 Ja: betaald: … uur

 Ja: onbetaald: … uur

 Nee

F8 *Heeft u de afgelopen 6 maanden wel eens gebruik van een andere vorm van zorg, (medisch, paramedisch, alternatief) die niet eerder in deze vragenlijst genoemd is?*

 Ja:

……………………………………………………………………………………………

……………………………………………………………………………………………

……………………………………………………………………………………………

……………………………………………………………………………………………

……………………………………………………………………………………………

……………………………………………………………………………………………

……………………………………………

 Nee

Welke van onderstaande medicamenteuze behandelingen gebruikt u (meerdere antwoorden mogelijk):

- Verapamil
  - Welke dosering?
    - 3 maal per dag 240 milligram
    - 2 maal per dag 240 milligram
    - 3 maal per dag 120 milligram
    - 2 maal per dag 120 milligram
    - Anders,…
    - Ik weet het niet
- Lithium
  - Welke dosering?
    - 1 maal per dag 1200 milligram / 2 maal per dag 600 milligram
    - Anders, …
    - Ik weet het niet
- Prednisolon
  - Welke dosering? ….. milligram per dag
    - Ik weet het niet
- Topamax (topiramaat)
  - Welke dosering
    - 1 maal per dag 25 milligram
    - 2 maal per dag 25 milligram
    - 1 maal per dag 25 milligram, 1 maal per dag 50 milligram
    - 2 maal per dag 50 milligram
    - Ik weet het niet
- Sandomigran (pizotifeen)

- Welke dosering?
  - 1 maal per dag 2,5 milligram
  - Weet ik het niet
- Frovatriptan
  - Welke dosering?
    - 1 maal per dag 2,5 milligram
    - 2 maal per dag 2,5 milligram
    - Weet ik het niet
- Ergotamine
  - Welke dosering?
    - 1 maal per dag 2 milligram
    - 2 maal per dag 2 milligram
    - 1 maal per dag 1 milligram en 1 maal per dag 2 milligram
    - Weet ik het niet
- Imigran
  - Hoeveel injecties **per week**? …
- Zuurstof
  - Hoeveel keer **per week** gebruikt u zuurstof?

Appendix C: Information letter and informed consent

Lange termijn resultaten van stimulatie van de achterhoofdzenuw voor medicamenteus onbehandelbare chronische cluster hoofdpijn
Lange termijn ICON studie

Patiënten informatie brief Augustus 2016

Versie 1.1

Geachte heer/mevrouw,

Wij vragen u om mee te doen aan een wetenschappelijk vervolg onderzoek naar het lange termijn effect van elektrische stimulatie van de achterhoofdzenuw bij medicamenteus onbehandelbare chronische cluster hoofdpijn. U beslist zelf of u wilt meedoen. Voordat u de beslissing neemt, is het belangrijk om meer te weten over het onderzoek. Lees deze informatiebrief daarom rustig door. Bespreek het eventueel met partner, vrienden of familie. U kunt ook de Algemene Brochure over wetenschappelijk onderzoek lezen. Daar staat veel algemene informatie over medisch-wetenschappelijk onderzoek in.

Hebt u na het lezen van deze informatie nog vragen, dan kunt u deze stellen aan de onderzoeker. Ook is er een onafhankelijke persoon, die veel weet van het onderzoek. Op bladzijde [4] vindt u de contactgegevens.

1. Wat is het doel van het onderzoek?

In het voorgaande onderzoek, waar u aan mee hebt gewerkt, is de werkzaamheid en veiligheid van stimulatie van de achterhoofdzenuw onderzocht bij patiënten met chronische clusterhoofdpijn bij wie medicijnen onvoldoende werkzaam zijn of te veel

bijwerkingen geven. Gezien deze toepassing nog op kleine schaal is toegepast, zijn we ook erg benieuwd naar de lange termijn resultaten en eventuele bijwerkingen.

2. Hoe wordt het onderzoek uitgevoerd?

Onderzoeksopzet
Nadat u heeft aangegeven dat u in principe wilt mee doen, zal u elk half jaar een vragenlijst via internet invullen. Dit duurt ongeveer 10 minuten. U zult via een email een herinnering van ons ontvangen. Ook zal er eenmalig 2 jaar na implantatie van de neurostimulator telefonisch contact met u gezocht worden door een van onze onderzoeksmedewerkers voor het afnemen van een vragenlijst. Dit duurt ongeveer 20 minuten.

Vragenlijst invullen
U kunt de inlogcode gebruiken welke u tijdens de ICON studie heeft gebruikt. Indien u deze niet meer heeft zal deze inlogcode opnieuw worden verstrekt. Met deze inlogcode kunt u de gegevens invullen op een speciale beveiligde internetsite.

3. Welke patiënten kunnen meedoen aan het onderzoek?

U heeft meegedaan aan de studie naar elektrische stimulatie van de achterhoofdzenuw bij medicamenteus onbehandelbare chronische cluster hoofdpijn (ICON). Ook als u de stimulator heeft verwijderd, kunt u deelnemen aan deze lange termijn vervolgstudie.

4. Wat wordt er van u verwacht?

U zult elk half jaar een digitale vragenlijst invullen. Ook zult u 3 keer telefonisch geïnterviewd worden. Onderhoudsmedicatie en aanvalsmedicatie mag u, volgens voorschrift van uw neuroloog, gebruiken. U mag in overleg met uw eigen neuroloog de medicatie tijdens dit onderzoek wijzigen.

5. Wat zijn mogelijke voor- en nadelen van deelname aan dit onderzoek?

Er zijn geen persoonlijke voordelen.

Mogelijke nadelen:
- het invullen van een vragenlijst elk half jaar
- Elk half jaar telefonisch of poliklinische contact, gedurende de eerste 2 jaar van dit onderzoek.

6. Wat gebeurt er als u niet wenst deel te nemen aan dit onderzoek?

U beslist zelf of u meedoet aan het onderzoek. Deelname is vrijwillig. Als u besluit niet mee te doen, hoeft u verder niets te doen. U hoeft niets te tekenen. U hoeft ook niet te zeggen waarom u niet wilt meedoen. U krijgt als patiënt gewoon de behandeling die u anders ook zou krijgen. Als u wel meedoet, kunt u zich altijd bedenken en stoppen. Dit kan ook tijdens het onderzoek.

7. Wat gebeurt er met uw gegevens?

Voor dit onderzoek is het nodig dat uw medische en persoonsgegevens worden verzameld en gebruikt. Elke proefpersoon krijgt een code die op de gegevens komt te staan. Uw naam en andere persoonsgegevens worden weggelaten.

Al uw gegevens blijven vertrouwelijk. Alleen de onderzoeker en de medewerkers van het onderzoeksteam weet/weten welke code u heeft. De sleutel voor de code blijft bij de onderzoeker. Ook in rapporten over het onderzoek wordt alleen die code gebruikt.

Sommige mensen mogen uw medische en persoonsgegevens inzien. Dit is om te controleren of het onderzoek goed en betrouwbaar uitgevoerd is. Algemene informatie hierover vindt u in de brochure ‘Medisch-wetenschappelijk onderzoek’.

Mensen die uw gegevens kunnen inzien zijn het onderzoeksteam, de veiligheidscommissie die het onderzoek in de gaten houdt, een controleur die voor de uitvoerder van het onderzoek werkt of die door de uitvoerder van het onderzoek is ingehuurd en de Inspectie voor de Gezondheidszorg. Zij houden uw gegevens geheim. Als u de toestemmingsverklaring ondertekent, geeft u toestemming voor het verzamelen, bewaren en inzien van uw medische en persoonsgegevens.

Wij willen uw gegevens 15 jaar bewaren. Vindt u het goed als wij uw gegevens bewaren? Wij kunnen daar later een ander onderzoek mee uitvoeren. Als er een nieuw onderzoek gaat beginnen, vragen wij u opnieuw om uw toestemming. U mag dan opnieuw aangeven of u wel of geen toestemming geeft voor het gebruik van uw gegevens.

8. Zijn er extra kosten/is er een vergoeding wanneer u besluit aan dit onderzoek mee te doen?

U krijgt geen vergoeding voor het invullen van de vragenlijsten.

9. Welke medisch-ethische toetsingscommissie heeft dit onderzoek goedgekeurd?

Toetsingscommissie [LUMC] heeft dit onderzoek goedgekeurd. Meer informatie over de goedkeuring vindt u in de Algemene brochure.

10. Wilt u verder nog iets weten?

De coördinator van het onderzoek kunt u bereiken via telefoon of email:

Mw. drs. P. Doesborg Afdeling neurologie, K5-104

Leiden Universitair Medisch Centrum

Leiden Nederland

Telefoon: 071-5261645

Email:p.g.g.doesborg@lumc.nl

Wilt u graag een onafhankelijk advies over meedoen aan dit onderzoek? Dan kunt u terecht bij een van de onafhankelijke artsen of ander deskundig persoon. De gegevens vindt u in deze brief:

Prof. Dr. J. G. Van Dijk

Neuroloog

Afdeling neurologie, K5-104

Leiden Universitair Medisch Centrum

Leiden Nederland

Telefoon: 071-5262895

19. Bijlagen:

- *Algemene brochure medisch-wetenschappelijk onderzoek met mensen*
- *verzekeringstekst*
- *lokale informatie*

Toestemmingsformulier
Brief Augustus 2016 Versie 1.1

Lange termijn onderzoek naar de stimulatie van de achterhoofdzenuw voor medicamenteus onbehandelbare chronische cluster hoofdpijn
Lange termijn-ICON studie

Ik heb de informatiebrief voor de proefpersoon gelezen. Ik kon aanvullende vragen stellen. Mijn vragen zijn voldoende beantwoord. Ik had genoeg tijd om te beslissen of ik meedoe.

Ik weet dat meedoen helemaal vrijwillig is. Ik weet dat ik op ieder moment kan beslissen om toch niet mee te doen. Daarvoor hoef ik geen reden te geven.

Ik geef toestemming om mijn huisarts te vertellen dat ik meedoe aan dit onderzoek.

Ik geef toestemming om de specialist(en) die mij behandelt te vertellen dat ik meedoe aan dit onderzoek.

Ik weet dat sommige mensen mijn gegevens kunnen zien. Die mensen staan vermeld in de Algemene brochure.

Ik geef toestemming om mijn gegevens te gebruiken, voor de doelen die in de informatiebrief staan.

Ik geef wel/geen* toestemming om gegevens nog maximaal 15 jaar na afloop van dit onderzoek te bewaren.

Ik vind het goed om aan dit onderzoek mee te doen.

Naam proefpersoon:

Handtekening: Datum : __ / __ / __

Ik verklaar hierbij dat ik deze proefpersoon volledig heb geïnformeerd over het genoemde onderzoek.

Als er tijdens het onderzoek informatie bekend wordt die de toestemming van de proefpersoon zou kunnen beïnvloeden, dan breng ik hem/haar daarvan tijdig op de hoogte.

Naam onderzoeker (of diens vertegenwoordiger):

Handtekening: Datum: __ / __ / __

Aanvullende informatie is gegeven door (indien van toepassing):

Naam:

Functie:

Handtekening: Datum: __ / __ / __

----

* Doorhalen wat niet van toepassing is.
