# Supplementary material for: A prospective open label 2–8 year extension of the randomised controlled ICON trial on the long-term efficacy and safety of occipital nerve stimulation in medically intractable chronic cluster headache
Source: eBioMedicine. 2023 Nov 25;98:104895. doi: 10.1016/j.ebiom.2023.104895 (PMC10755111; doi:10.1016/j.ebiom.2023.104895)
Supplement: PubMed table [file mmc3.docx]

| First name | Surname |
| --- | --- |
| Michel D | Ferrari |
| Leopoldine A | Wilbrink |
| Ilse F | De Coo |
| Patty G | Doesborg |
| Eveline C | Bartels |
| Erik W | Van Zwet |
| Frank J P M | Huygen |
| Wim M | Mulleners |
| Erkan | Kurt |
| Robert T M | Van Dongen |
| Onno P M | Teernstra |
| Peter J | Koehler |
| Geert H | Spincemaille |
| Frank | Wille |
| Katja | Burger |
| Joost | Haan |
| Emile G M | Couturier |
| Jan Willem | Kallewaard |
| Peter H | Veltink |
| R | Buschman |
