# Supplementary material for: A prospective open label 2–8 year extension of the randomised controlled ICON trial on the long-term efficacy and safety of occipital nerve stimulation in medically intractable chronic cluster headache
Source: eBioMedicine. 2023 Nov 25;98:104895. doi: 10.1016/j.ebiom.2023.104895 (PMC10755111; doi:10.1016/j.ebiom.2023.104895)
Supplement: ICON group [file mmc4.docx]

**Appendix 1**

**ICON study group**

Investigators are listed by center

1. Leiden University Medical Center - M.D. Ferrari (Chair), L.A. Wilbrink, I. F. de Coo, P.G.G. Doesborg, E.C. Bartels, E.W. van Zwet
2. Erasmus Medical Center - F.J.P.M. Huygen (Vice Chair)
3. Canisius Wilhelmina Hospital - W. Mulleners, E. Kurt
4. Radboud Medical Center - R.T.M. van Dongen
5. Zuyderland Hospital - O.P. Teernstra, P.J.J. Koehler, G.H. Spincemaille
6. Diakonessenhuis Zeist - F. Wille
7. Alrijne Hospital - K. Burger, J. Haan
8. Boerhaave Medical Center - E.G.M. Couturier
9. Rijnstate Hospital Arnhem - J.W. Kallewaard
10. University of Twente – Peter H. Veltink
11. Medtronic BV - R. Buschman
